# Cell Type- and Layer-Specific Muscarinic Potentiation of Excitatory Synaptic Drive onto Parvalbumin Neurons in Mouse Prefrontal Cortex

**DOI:** 10.1523/ENEURO.0208-18.2018

**Published:** 2018-11-15

**Authors:** Tatiana B. Tikhonova, Takeaki Miyamae, Yelena Gulchina, David A. Lewis, Guillermo Gonzalez-Burgos

**Affiliations:** 1 Translational Neuroscience Program, Department of Psychiatry, University of Pittsburgh School of Medicine, Pittsburgh, PA 15261

**Keywords:** Prefrontal cortex, acetylcholine, EPSC, parvalbumin, interneuron, muscarinic receptor

## Abstract

Cholinergic neuromodulation is thought to shape network activity in the PFC, and thus PFC-dependent cognitive functions. ACh may modulate the activity of parvalbumin-positive (PV^+^) neurons, which critically regulate cortical network function. However, the mechanisms of cholinergic regulation of PV^+^ neuron activity, and particularly of the basket cell (BC) versus chandelier cell (ChC) subtypes, are unclear. Using patch clamp recordings in acute slices, we examined the effects of the ACh receptor (AChR) agonist carbachol on the excitatory synaptic drive onto BCs or ChCs in layers 2 to 6 of mouse PFC. Carbachol increased the frequency and amplitude of spontaneous EPSCs (sEPSCs) recorded from PV^+^ BCs in layers 3-6, but not in BCs from layer 2. Moreover, carbachol did not change the sEPSCs in ChCs, which were located exclusively in layer 2. The potentiation of sEPSCs in layers 3-6 BCs was prevented by the Na^+^ channel blocker tetrodotoxin and was abolished by the M1-selective muscarinic AChR antagonist pirenzepine. Thus, carbachol potentiates the activity-dependent excitatory drive onto PV^+^ neurons via M1-muscarinic AChR activation in a cell type- and layer-specific manner. In current clamp recordings with synaptic transmission blocked, carbachol directly evoked firing in deep layer pyramidal neurons (PNs). In contrast, carbachol elicited deep layer BC firing indirectly, via glutamate-mediated synaptic drive. Our data suggest that ACh powerfully regulates PFC microcircuit function by facilitating the firing of PNs that synaptically recruit deep layer PV^+^ BC activity, possibly shaping the patterns of network activity that contribute to cognitive function.

## Significance Statement

Cholinergic neuromodulation and parvalbumin-positive (PV^+^) neurons may be essential for regulation of PFC network activity. To determine whether cholinergic input modulates PFC network function via PV^+^ neurons, we examined the effects of the cholinergic agonist carbachol on the excitatory synaptic drive onto PV^+^ neurons in mouse PFC. Carbachol, via M1 muscarinic ACh receptor activation, potentiated the excitatory synaptic currents onto PV^+^ basket cells (BCs) in deep cortical layers, but not onto superficial layer BCs or chandelier cells (ChCs). Carbachol directly elicited firing in deep layer pyramidal neurons (PNs) but BC activity was recruited indirectly, via synaptic glutamate-mediated excitatory drive. Our data suggest that cholinergic neuromodulation contributes to PFC-dependent cognitive function recruiting PV^+^ neuron activity in a cell type- and layer-specific manner.

## Introduction

Regulation of cortical network activity by ACh plays a crucial role in cognitive function (Ballinger et al., 2016). For example, PFC activity is strongly modulated by cholinergic input (Picciotto et al., 2012; Arnsten and Wang, 2016). In addition, ACh depletion in the PFC (Croxson et al., 2011), or intra-PFC infusion of ACh receptor (AChR) antagonists (Howe et al., 2017), disrupt performance in cognitive tasks. Despite this crucial role of ACh, the mechanisms of cholinergic modulation of PFC network activity remain poorly understood.

Muscarinic receptor (mAChR) activation increases PFC pyramidal neuron (PN) firing via direct depolarization and/or enhancing the intrinsic excitability of PNs (Obermayer et al., 2017; Baker et al., 2018; Radnikow and Feldmeyer, 2018). Many of the ACh effects on PNs, such as the excitatory mAChR response (McCormick and Prince, 1986; Gulledge et al., 2009), are layer dependent, specifically being stronger in deep layers (Obermayer et al., 2017; Radnikow and Feldmeyer, 2018). Deep layer PNs also show stronger nicotinic AChR-mediated responses (Poorthuis et al., 2013; Hedrick and Waters, 2015), which may be dependent on cortical region (Hedrick and Waters, 2015).

Relatively less is known about the effects of ACh on interneurons, particularly of the parvalbumin-positive (PV^+^) class (Muñoz and Rudy, 2014), which provide strong perisomatic inhibition onto PNs and are crucial for regulation of cortical network activity (Hu et al., 2014). Furthermore, the effects of ACh have not been compared between the two main subtypes of PV^+^ neurons, basket cells (BCs) and chandelier cells (ChCs), which target the perisomatic PN membrane. Whereas BCs innervate the soma and proximal dendrites, ChCs target the axon initial segment (Somogyi et al., 1998; DeFelipe et al., 2013). Moreover, ChCs and BCs both display a fast-spiking phenotype *in vitro* (Hu et al., 2014) but exhibit different firing dynamics *in vivo* (Zhu et al., 2004; Klausberger and Somogyi, 2008; Massi et al., 2012; Viney et al., 2013). Thus, regulation of network activity by PV^+^ neurons seems to involve a division of labor between ChCs and BCs, but whether cell type-specific neuromodulation contributes to the different roles played by ChCs and BCs is unclear.

Given that AChR stimulation increases PN firing, it may also enhance the excitatory synaptic drive onto PV^+^ neurons, thus being essential for the regulation of PFC network activity via PV^+^ neuron-mediated inhibition (Picciotto et al., 2012). To test whether AChR stimulation modulates the excitatory drive onto PV^+^ neurons, we assessed the effects of the AChR agonist carbachol on spontaneous EPSCs (sEPSCs) recorded from PV^+^ neurons in acute slices from mouse PFC. Consistent with previous findings that stimulation of M1 mAChRs facilitates deep layer PN firing (Carr and Surmeier, 2007), we found that carbachol increased the excitatory drive onto PV^+^ neurons in layers 3-6, via an effect prevented by an M1-selective mAChR antagonist (pirenzepine) or by tetrodotoxin, a Na^+^ channel blocker that inhibits action potential firing. In contrast, carbachol did not have effects on the excitatory drive onto PV^+^ neurons located in layer 2. Analysis of the morphology of the recorded neurons revealed that all layers 3-6 PV^+^ neurons were BCs, whereas among the layer 2 PV^+^ cells, ∼50% were ChCs and ∼50% were BCs. Current clamp recordings showed that mAChR activation can directly evoke firing in layer 5 PNs, but elicits layers 3-6 BC firing indirectly, via glutamate-mediated synaptic input. Thus, our data show that mAChRs enhance the excitatory drive onto PV^+^ neurons in a cell type- and layer-specific manner, selectively recruiting the activity of BCs in deep layers of PFC.

## Materials and Methods

All animal procedures were performed in accordance with the guidelines of the National Institutes of Health Guide for Care and Use of Laboratory Animals and were approved by our institution’s Animal Care and Use Committee.

### Slice preparation

The experiments were performed using G42 mice of both sexes (The Jackson Laboratory, stock number 007677; RRID:IMSR_JAX:007677) which express green fluorescent protein (GFP) exclusively in PV^+^ interneurons (Chattopadhyaya et al., 2004). The mice (aged postnatal days 23–42) were quickly decapitated under deep isoflurane anesthesia, and the brain was removed and placed in ice-cold slicing solution containing: 120 mM choline chloride, 2.5 mM KCl, 1.2 mM Na_2_HPO_4_, 25 mM NaHCO_3_, 20 mM glucose, 1.3 mM ascorbate, 2.4 mM pyruvate, 7 mM MgCl_2_, and 0.5 mM CaCl_2_; pH 7.3–7.4, and continuously bubbled with 95% O_2_-5% CO_2_. In the experiments testing the effects of tetrodotoxin, atropine, or pirenzepine, the slices were prepared in a slicing solution containing: 200 mM sucrose, 15 mM NaCl, 1.9 mM KCl, 1.2 mM Na_2_HPO_4_, 33 mM NaHCO_3_, 10 mM glucose, 2 mM kynurenic acid, 6 mM MgCl_2_, and 0.5 mM CaCl_2_; pH 7.3–7.4, continuously bubbled with 95% O_2_-5% CO_2_. Coronal brain slices (300 µm thick) were prepared from the frontal cortex using a vibrating microtome (VT1000S or VT1200S, Leica Microsystems) and incubated for 5 min at 36°C in oxygenated (95% O_2_-5% CO_2_) artificial CSF (ACSF; pH 7.4) solution containing: 125 mM NaCl, 2.5 mM KCl, 1.25 mM Na_2_HPO_4_, 10 mM glucose, 25 mM NaHCO_3_, 0.4 mM ascorbate, 1 mM MgCl_2_, and 2 mM CaCl_2_. The slices were allowed to equilibrate at room temperature for at least 30 min before they were transferred to the recording chamber. The potentiation of sEPSC frequency by 20 µM carbachol did not differ between layer 3-6 BCs recorded from slices prepared with choline-based slicing solution (baseline: 9.3 ± 7.7 Hz; carbachol: 33.1 ± 26 Hz, *n* = 7) versus the sucrose-based slicing solution [baseline: 14.1 ± 7.4 Hz; carbachol: 32.7 ± 8.6 Hz, *n* = 5; carbachol effect: *F*_(1,10)_ = 18.077, *p* = 0.00168; Slicing solution effect *F*_(1,10)_ = 0.255, *p* = 0.6248, two-way repeated measures (RM) ANOVA].

### Electrophysiological recordings and data analysis

Recordings were performed in a submersion chamber superfused at a rate of 5 ml/min (Hajos et al., 2009) with oxygenated ACSF at 30–32°C, containing 10 µM SR-95531 (gabazine), a GABA_A_ receptor antagonist, to block inhibitory postsynaptic currents. In some experiments 10 µM 6-cyano-7-nitroquinoxaline-2,3-dione (CNQX), an AMPA/kainate antagonist, was added to block excitatory postsynaptic currents. We found that the fast perfusion rate significantly improved the stability of the effects of carbachol, as reported previously (Hajos et al., 2009). Whole-cell recordings were obtained from PV^+^ neurons in the medial frontal cortex [infralimbic (IL), prelimbic (PL), and anterior cingulate (AC) regions]. Neurons were identified by the presence of GFP fluorescence, using Olympus or Zeiss microscopes equipped with epifluorescence, infrared illumination, differential interference contrast, and a CCD video camera (EXi Aqua, Q-Imaging). Pipettes pulled from borosilicate glass (resistance: 3–6 MΩ) were filled with the following solution: 120 mM potassium gluconate, 10 mM KCl, 10 mM HEPES, 0.2 mM EGTA, 4.5 mM MgATP, 0.3 mM NaGTP, 14 mM sodium phosphocreatine; the pH was adjusted to 7.2–7.4 using KOH. Biocytin (0.5%) was added to the pipette solution for later morphologic identification. Recordings were obtained with Multiclamp 700B or 700A amplifiers (Molecular Devices). Signals were low-pass filtered at 6 kHz and digitized at 10 or 20 kHz using a Power 1401 data acquisition interface (Cambridge Electronic Design). Data acquisition was performed using Signal 5 software (Cambridge Electronic Design), running custom-made scripts.

#### Voltage clamp

PV^+^ neurons were recorded while holding their membrane potential at –75 mV and in the presence of gabazine. In these conditions, the EPSCs recorded from PV^+^ neurons were entirely blocked by the AMPA receptor antagonist CNQX (data not shown). The pipette capacitance (Cp) was compensated and the series resistance (Rs) was continuously monitored, but Rs compensation was not used. Only recordings with an initial Rs < 15 MΩ were used for analysis. The stability of Rs was measured using the current transient evoked by a 50 ms–5 mV voltage step, delivered every 5 s, and recordings were not used for data analysis if the Rs changed by >15% of the initial value.

For each recorded PV^+^ neuron, sEPSCs were detected in 20-s time windows, every min, for ≥ 5 min before the application of carbachol, during carbachol application, and through 15 min of washout. EPSCs were detected and analyzed using Mini Analysis software (Synaptosoft), with an amplitude threshold of 4–6 pA, area threshold of 4 pA/ms and setting the average baseline before EPSC onset to 2–10 ms. The detected sEPSCs were visually inspected and used to estimate the mean peak sEPSC amplitude and sEPSC frequency. The mean sEPSC frequency was calculated for each 20-s time window analyzed, from the number of sEPSCs detected in each time window and expressed in Hz. The mean sEPSC amplitude was calculated, for each recorded neuron, as the average of the amplitudes of all sEPSCs detected, during control, carbachol or washout periods.

#### Current clamp

Cells included in this study had an initial resting membrane potential of −60 to −80 mV. Current clamp recordings from PV^+^ neurons and pyramidal cells in layers 3-6 were conducted in the continuous presence of the GABA_A_ receptor antagonist gabazine (10 µM) and the glutamate receptor antagonist CNQX (10 or 20 µM), except for some recordings in which CNQX was omitted, as indicated. The Rs and Cp were monitored and cancelled using the bridge balance and capacitance neutralization circuits. The rheobase was estimated as the smallest current step amplitude eliciting at least one action potential in three repetitions of that current step. Subsequently, depolarizing current steps (500 ms duration) were injected in 10 pA increments from 10 pA below rheobase to +30 or +40 pA above rheobase continuously, throughout the duration of the experiment. Resting membrane potential was determined by averaging the values measured from a 50 ms window placed before a hyperpolarizing current step (–50 pA, 50 ms) used to monitor stability of the input resistance and membrane time constant. The frequency of action potentials elicited by carbachol independent of current injection was measured in the 200- to 500-ms window preceding the injected current steps, as indicated in Figures 10, 11. The depolarizing current step injection was used to test the possibility that carbachol increases the response to the excitatory current steps without directly depolarizing the cells’ membrane potential. Baseline was collected for ≥5 min before bath application of carbachol (20 µM) for 5 min. In some current clamp recordings from PV^+^ cells or PNs, atropine (10 µM) or pirenzepine (1 µM) were continuously bath applied.

### Histologic processing and morphologic reconstruction of biocytin-filled neurons

The PV^+^ neurons filled with 0.4–0.5% biocytin during recordings were visualized and reconstructed using standard procedures. Briefly, after recordings, the slices were immersed in 4% p-formaldehyde in 0.1 M PBS for 24–72 h at 4°C. The slices were stored at −80°C in cryo-protection solution (33% glycerol, 33% ethylene glycol, in 0.1 M PBS) until processed. To visualize biocytin, the slices were resectioned at 60 μm, incubated with 1% H_2_O_2_, and immersed in blocking serum containing 0.5% Triton X-100 for 2–3 h at room temperature. The tissue was then rinsed and incubated with the avidin–biotin–peroxidase complex (1:100; Vector Laboratories) in PBS for 4 h at room temperature. Sections were rinsed, stained with the Nickel-enhanced 3,3′-diaminobenzidine chromogen, mounted on gelatin-coated glass slides, dehydrated, and coverslipped. Three-dimensional reconstructions were performed using the Neurolucida tracing system (MBF Bioscience). Here we report data on the morphologic reconstructions of PV^+^ neurons for which we tested the effects of carbachol on sEPSCs.

ChCs are morphologically defined by an axonal arbor with abundant cartridges, short rows of boutons aligned vertically and connected by axonal segments of thin diameter, which reflect targeting of the axon initial segment of neighbor pyramidal cells (DeFelipe et al., 2013). ChC axons typically display between two and nine boutons per cartridge (Inan et al., 2013). In contrast to ChCs, the axons of BCs lack cartridges, reflecting their targeting of soma and dendrites, but not the axon initial segment, of their postsynaptic pyramidal cells (DeFelipe et al., 2013).

### Laminar location of the somata of the recorded PV^+^ neurons

**Figure 1.**
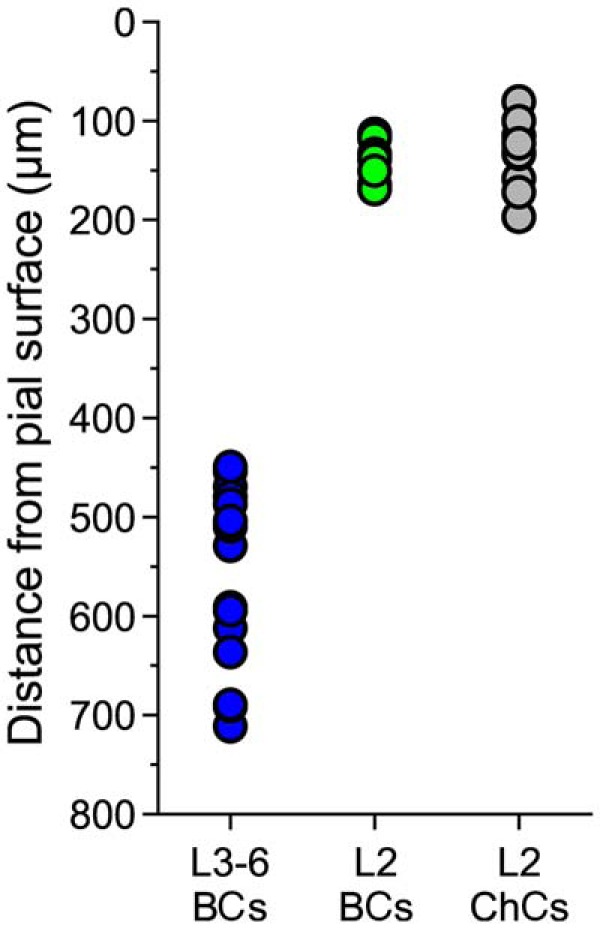
Distance between the pial surface and the PV^+^ cell somata for BCs of layers 3-6 (L3-6 BCs), for BCs of layer 2 near the border with layer 1 (L2 BCs) and for ChCs of layer 2 near the border with layer 1 (L2 ChCs).

GFP-positive (GFP^+^) PV^+^ neurons were selected for recording based on their laminar location as follows. Due to the low neuron density present in layer 1, the location of the layer 1-2 border was easily distinguished under differential interference contrast imaging of the slices in the recording chamber. To record from PV^+^ neurons of ChC subtype, we targeted for recording GFP^+^ cells in superficial layer 2 near the border with layer 1, since previous studies showed that ChCs are abundant at this laminar location, where BCs are abundant as well (Woodruff et al., 2011; Miyamae et al., 2017). To target for recording PV^+^ cells in layers 3-6, first we estimated the thickness of layer 1 using low magnification images of the slices in the recording chamber (measured in 10 slices, the layer 1 thickness was, mean ± SEM, 141 ± 39 µm, range: 90–205 µm). In the rodent PFC, the border between layers 2 and 3 is less well defined than the layer 1/2 border and cannot be reliably identified in the slices during the electrophysiological recording experiments. However, the average thickness of layer 2 is similar to the thickness of layer 1 (Gabbott et al., 1997; Ding et al., 2001; Van De Werd et al., 2010). Therefore, using the location of the layer 1/2 border in each slice, to record from PV^+^ cells in layers 3-6, we targeted GFP^+^ cells with somata located at a distance from the pial surface greater than ∼2.5 times the thickness of layer 1. Using this procedure, we did not target for recordings GFP^+^ neurons with soma located in deep layer 2 and superficial layer 3 (Fig. 1), where all PFC PV^+^ neurons are BCs (Miyamae et al., 2017; Pafundo et al., 2018) and ChCs are absent (Taniguchi et al., 2013). The laminar position of the deep layer PV^+^ neurons was determined using approximate laminar boundaries, as follows. In the rodent PFC, the border between layers 3 and 5 is located at approximately half the distance between the pia and the white matter, while the border between layers 5 and 6 is located at approximately half the distance between the layers 3-5 border and the white matter border (Gabbott et al., 1997; Ding et al., 2001; Van De Werd et al., 2010).

### Statistical analysis

The data were expressed as mean ± SEM. The significance of the effects of carbachol on sEPSC frequency or amplitude was assessed using one-way RM ANOVA, or one-way RM mixed model ANOVA, and in some cases paired *t* tests. To assess the significance of differences between group means, we first calculated the residuals for each variable, and performed Shapiro–Wilk tests of normality of the residuals. When necessary depending on the *p* value of the Shapiro–Wilk test, we employed natural logarithm transformation of the data. When the Shapiro–Wilk tests were significant before or after log transformation, we used rank transformation of the data. The results of the Shapiro–Wilk tests are reported, for data illustrated in the figures, in the statistics table (Table 1), and otherwise in the text. In all figures, values are shown without transformation. The statistical analysis was performed using SPSS 20 (IBM Corp.).

**Table 1.** Statistics table

Data	Data Structure (Shapiro–Wilk test, *p* value)	Statistical test	*p* value
Figure 2*E*	0.016^*^	One-way RM ANOVA *F*_(2,13.01)_ = 27.26^**^	<0.001^***^
Figure 2*F*	0.721	One-way RM ANOVA *F*_(2,12.89)_ = 5.49^**^	0.019
Figure 3*C*	0.146	One-way RM ANOVA *F*_(2,16)_ = 0.79	0.469
Figure 3*D*	0.988	One-way RM ANOVA *F*_(2,16)_ = 5.54	0.017
Figure 4*C*	0.080^*^	One-way RM ANOVA *F*_(2,19.5)_ = 2.53^**^	0.106
Figure 4*D*	0.648	One-way RM ANOVA *F*_(2,15.4)_ = 2.89^**^	0.086
Figure 6*B,C*	0.0795^*^	Two-way ANOVA, Cell type: *F*_(2,17)_ = 7.647, Area: *F*_(2,17)_ = 1.101 Interaction: *F*_(4,17)_ = 0.191	*p* = 0.00428; *p* = 0.355; *p* = 0.939
Figure 7*C*	0.357	*F*_(2,20)_ = 4.634	*p* = 0.022
Figure 7*D*	0.178	*F*_(2,20)_ = 1.281	*p* = 0.299
Figure 8*C*	0.101^*^	One-way RM ANOVA *F*_(2,16.46)_ = 0.81^**^	0.461
Figure 8*D*	0.517	One-way RM ANOVA *F*_(2,16.11)_ = 0.52^**^	0.602
Figure 9*C*	0.0485^*^	One-way RM ANOVA *F*_(2,27.3)_ = 0.565^**^	*p* = 0.575^***^
Figure 9*D*	0.004^*^	One-way RM ANOVA *F*_(2,27.3)_ = 0.0095^**^	*p* = 0.991^***^
Figure 10*C*	0.787	Paired sample *t* test *t*_(6)_ = –5.041	0.0023
Figure 10*E*	0.458	One-way RM ANOVA *F*_(2,8)_ = 8.555	0.01
Figure 11*B*	0.1707	One-way RM ANOVA *F*_(1,8)_ = 6.579	0.033
Figure 11*D*	0.0439^*^	One-way RM ANOVA *F*_(2,8)_ = 1.500	0.280^***^

* Shapiro–Wilk test performed on the residuals of the log-transformed data.

** Mixed model RM ANOVA.

*** RM ANOVA was performed using the data rank transformation (see Materials and Methods, Statistical analysis).

## Results

To assess the effects of AChR activation on excitatory drive onto PV^+^ neurons, we recorded sEPSCs from GFP^+^ cells in the PFC of G42 mice, which express GFP selectively in PV^+^ neurons (Chattopadhyaya et al., 2004; Buchanan et al., 2012; Sippy and Yuste, 2013). Previous studies reported that whereas PV^+^ BCs are located across all layers, ChCs are concentrated or exclusively located near the border between layers 1 and 2 (Woodruff et al., 2011; Miyamae et al., 2017). Thus, we targeted for recording GFP^+^ cells with soma located in deep layer 3 to layer 6 (L3-6 cells), or in layer 2 near the border with layer 1 (L2 cells). The GFP^+^ neurons were filled with biocytin during recordings to determine the morphologic subtype of each recorded cell. We examined a total of 82 GFP^+^ neurons: 63 L3-6 cells, and 19 L2 cells. As reported in earlier studies (Woodruff et al., 2011; Miyamae et al., 2017), ChCs were found exclusively near the border between layers 1 and 2, where they comprised nearly half (10/19) of the L2 cells, as the rest of L2 GFP^+^ cells (9/19) were BCs. On the other hand, all L3-6 GFP^+^ neurons were BCs, consistent with a previous study of mouse PFC (Miyamae et al., 2017).

**Figure 2.**
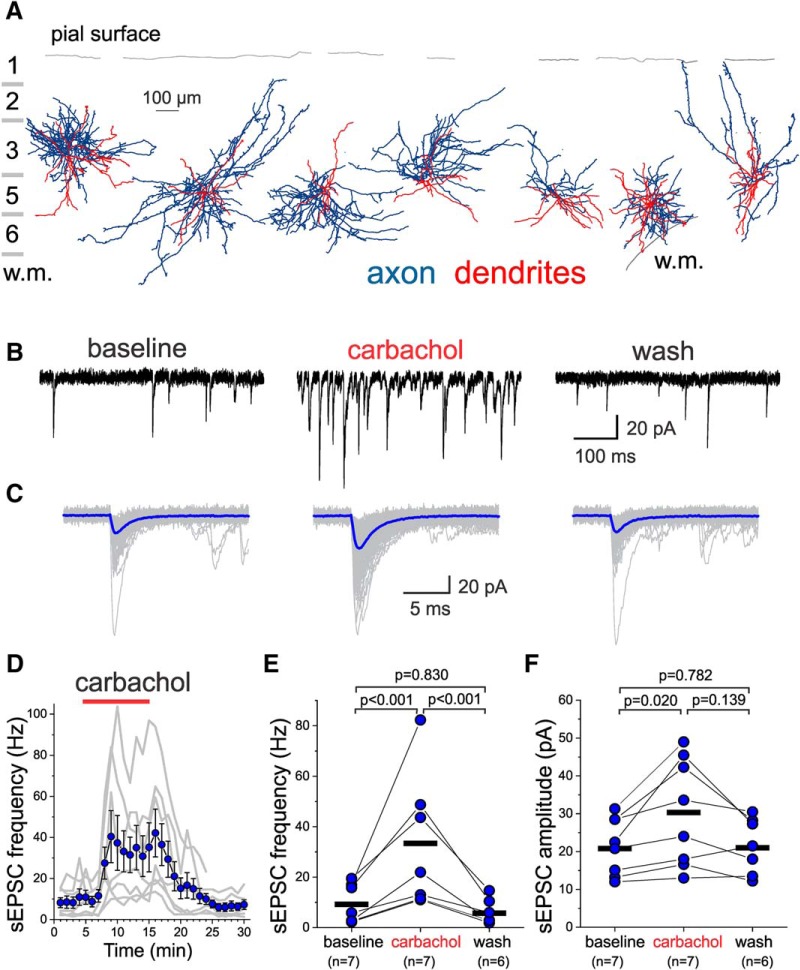
Effects of the AChR agonist carbachol on sEPSCs recorded from layers 3-6 PV^+^ BCs. ***A***, Examples of the morphology of layers 3-6 PV^+^ BCs, with their somata aligned relative to the pial surface (gray line), and displayed relative to the approximate boundaries of cortical layers. The dendritic tree is shown in red and the axonal arbor in blue. For a BC on the right part of this panel, the gray line at the bottom shows the border between layer 6 and white matter (w.m.). ***B***, Examples of sEPSCs recorded from a layer 5 BC before (baseline), during 20 µM carbachol application (carbachol), and after washout (wash). ***C***, Examples of individual sEPSCs (gray traces) are shown superimposed aligned by their rise time, together with an average sEPSC (blue traces) obtained for each condition indicated in ***B***. Approximately 200 consecutive sEPSCs were averaged. ***D***, Time-course plot illustrating the effects of a 10-min application of 20 µM carbachol on sEPSC frequency. The gray lines show the data for individual L3-6 BCs. The symbols show mean ± SEM (*n* = 7). The sEPSC frequency was measured in 20 s time windows every 60 s. ***E***, Carbachol had significant effects on sEPSC frequency when estimated at the last 20 s before carbachol application (baseline), the last 20 s before beginning of washout, and after ≥10 min of washout (One-way RM mixed model ANOVA, *F*_(2,13.01)_ = 27.26, *p* < 0.001, shown in the figure are the *p* values for Sidak-corrected *post hoc* pairwise comparisons). The black horizontal bars indicate the mean value for each sample. ***F***, Carbachol had significant effects on sEPSC amplitude when estimated between the last 20 s before carbachol application (baseline), the last 20 s before beginning of washout, and after ≥10 min of washout (one-way RM ANOVA mixed model, *F*_(2,12.89)_ = 5.486, *p* = 0.019; shown in figure are the results of Sidak-corrected, *post hoc* pairwise comparisons). The black horizontal bars indicate the mean value for each sample.

The effects of AChR stimulation on excitatory drive were assessed in the following manner: after ≥5 min of baseline sEPSC recordings, carbachol (20 µM) was perfused for 10 min, followed by 15 min of washout. Figure 2*A* shows morphologic reconstructions of the axonal arbor and dendritic tree of the seven L3-6 BCs for which we tested the effects of carbachol on sEPSCs. Carbachol caused a marked increase in sEPSC frequency in L3-6 BCs (Fig. 2*B*), which was accompanied by an increase in the mean sEPSC amplitude (Fig. 2*C*). The effect of carbachol developed within 2–3 min of application and was rapidly reversed by washout (Fig. 2*D*). Both the increases in sEPSC frequency (Fig. 2*E*) and sEPSC amplitude (Fig. 2*F*) by carbachol were significant, and reversed significantly with washout (one-way RM mixed model ANOVA, sEPSC frequency: *F*_(2,13.01)_ = 27.26, *p* < 0.001, sEPSC amplitude: *F*_(2,12.89)_ = 5.486, *p* = 0.019).

**Figure 3.**
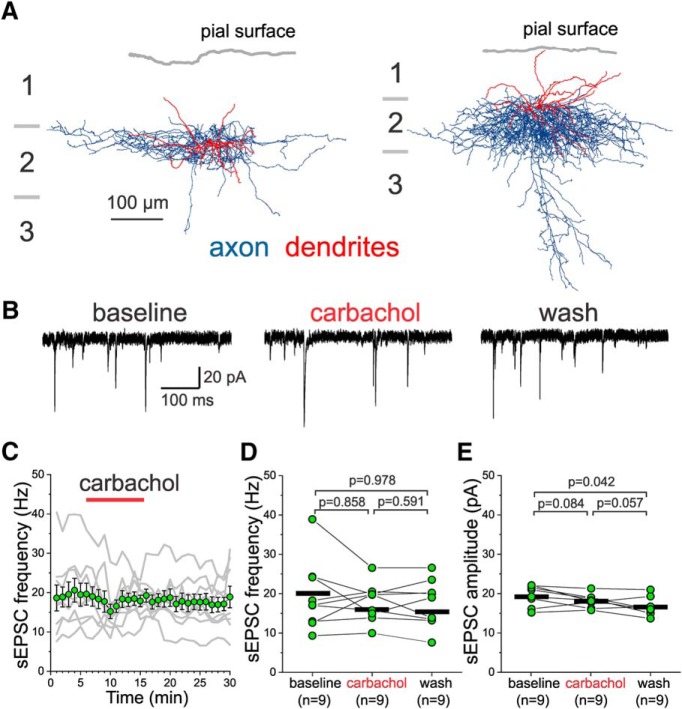
Effects of carbachol on sEPSCs recorded from layer 2 PV^+^ BCs with soma near the border with layer 1. ***A***, Examples of the morphology of layer 2 PV^+^ BCs, with their somata aligned relative to the pial surface (gray line). The dendritic tree is shown in red and the axonal arbor in blue. Note that the BC dendrites are nearly evenly distributed between layers 1 and 2, and that the distal tips of the BC dendrites in layer 1 mostly end far from the pial surface. ***B***, Examples of sEPSCs recorded from a layer 2 BC before (baseline), during 20 µM carbachol application (carbachol), and after washout (wash). ***C***, Time-course plot illustrating the effects of a 10 min application of 20 µM carbachol on sEPSC frequency. The gray lines show the data for individual BCs. The symbols show mean ± SEM, *n* = 9. The sEPSC frequency was measured in 20 s time windows every 60 s. ***D***, Carbachol did not have significant effects on sEPSC frequency when estimated 20 s before carbachol application (baseline), the last 20 s before beginning of washout, and after ≥10 min of washout (one-way RM ANOVA, *F*_(2,16)_ = 0.794, *p* = 0.469; shown in figure are the results of Sidak-corrected, *post hoc* pairwise comparisons). The black horizontal bars indicate the mean value for each sample. ***E***, Carbachol did not significantly increase the sEPSC amplitude when estimated 20 s before carbachol application (baseline), the last 20 s before beginning of washout, and after ≥10 min of washout (one-way RM ANOVA, *F*_(2,16)_ = 5.541, *p* = 0.017; shown in figure are the results of Sidak-corrected, *post hoc* pairwise comparisons). The black horizontal bars indicate the mean value for each sample.

Figure 3*A* shows reconstructions of the axonal arbor and dendritic tree of two representative examples of the L2 BCs (*n* = 9) for which we tested the actions of carbachol on sEPSCs (Fig. 3*B*). In contrast to the effects observed in L3-6 BCs, carbachol did not significantly increase the sEPSC frequency (Fig. 3*C*,*D*) or sEPSC amplitude (Fig. 3*E*) in L2 BCs, whereas a small but significant decline in sEPSC amplitude was observed between baseline and washout periods (one-way RM ANOVA, sEPSC frequency: *F*_(2,16)_ = 0.794, *p* = 0.469, sEPSC amplitude: *F*_(2,16)_ = 5.541, *p* = 0.017).

**Figure 4.**
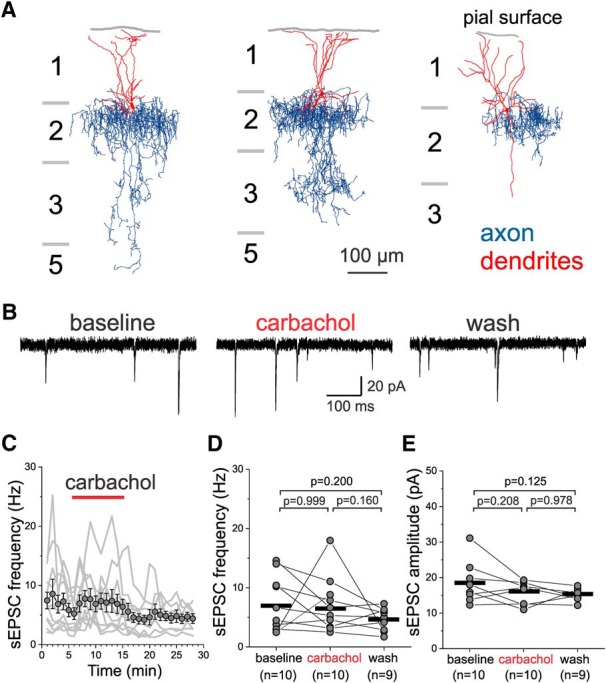
Effect of carbachol on sEPSCs recorded from layer 2 PV^+^ ChCs with soma near the border with layer 1. ***A***, Examples of the morphology of ChCs with soma near the border with layer 1. The dendritic tree is shown in red and the axonal arbor in blue. Note that ChC dendrites mostly project onto layer 1 and that the distal tips of the dendrites end near the pial surface. ***B***, Examples of sEPSCs recorded from a L2 ChC before (baseline), during 20 µM carbachol application (carbachol), and after washout (wash). ***C***, Time-course plot illustrating the effects of a 10 min application of 20 µM carbachol on sEPSC frequency in ChCs. The gray lines show the data for individual ChCs. The symbols show mean ± SEM, *n* = 10. The sEPSC frequency was measured in 20 s time windows every 60 s. ***D***, Carbachol did not have significant effects on sEPSC frequency when estimated 20 s before carbachol application (baseline), the last 20 s before beginning of washout, and after ≥10 min of washout; one-way RM mixed model ANOVA, *F*_(2,19.5)_ = 2.53, *p* = 0.106; shown in figure are the results of Sidak-corrected, *post hoc* pairwise comparisons). The black horizontal bars indicate the mean value for each sample. ***E***, Carbachol did not have significant effects on sEPSC amplitude when estimated 20 s before carbachol application (baseline), the last 20 s before beginning of washout, and after ≥10 min of washout (one-way RM mixed model ANOVA, *F*_(2,15.4)_ = 2.89, *p* = 0.086; shown in figure are the results of Sidak-corrected, *post hoc* pairwise comparisons). The black horizontal bars indicate the mean value for each sample.

Figure 4*A* shows reconstructions of the axonal arbor and dendritic tree of three representative examples of the L2 ChCs (*n* = 10) tested with carbachol during sEPSC recordings. In L2 ChCs, carbachol application did not change the sEPSCs (Fig. 4*B*), in contrast to the increase observed in L3-6 BCs. Neither the sEPSC frequency (Fig. 4*C*,*D*) nor sEPSC amplitude (Fig. 4*E*) were significantly affected by carbachol in L2 ChCs (one-way RM mixed model ANOVA, sEPSC frequency: *F*_(2,19.5)_ = 2.53, *p* = 0.106, sEPSC amplitude: *F*_(2,15.4)_ = 2.89, *p* = 0.086). The data showing that carbachol increases the sEPSC frequency and amplitude in L3-6 BCs but not in L2 BCs or L2 ChCs suggest that AChR stimulation increases the excitatory drive onto PV^+^ neurons in a cell-type and layer-specific manner.

**Figure 5.**
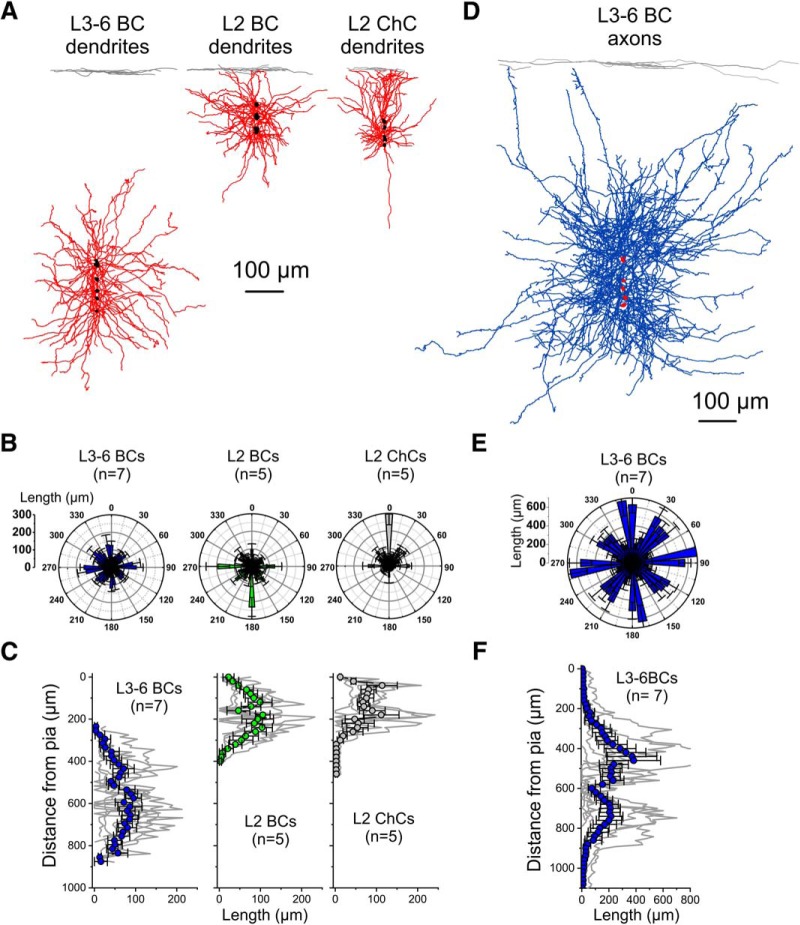
Quantitative morphometric analysis of PV^+^ neuron dendritic trees and axonal arbors. ***A***, Reconstructions of the dendritic tree of individual PV^+^ cells are shown superimposed and aligned, in the *y*-axis, relative to the pial surface and centered, in the *x*-axis, by the cell body. The somata are indicated in black. ***B***, Polar histograms of the distribution of the PV^+^ neuron dendrite length, relative to the soma. ***C***, Distribution of the PV^+^ neuron dendrite length, as a function of distance from the pial surface. The symbols show mean ± SEM. ***D***, Reconstructions of the axonal arbor of individual PV^+^ L3-6 BCs are shown superimposed and aligned, in the *y*-axis, relative to the pial surface and centered, in the *x*-axis, by the cell body. The somata are indicated in red. ***E***, Polar histogram of the distribution of the PV^+^ L3-6 BC axon length, relative to the soma. ***F***, Distribution of the PV^+^ L3-6 BC axon length, as a function of distance from the pial surface. The symbols show mean ± SEM.

The marked contrast between the strong potentiation by carbachol of excitatory synaptic input in L3-6 BCs versus the absence of significant potentiation in L2 BCs or L2 ChCs prompted us to assess if these differences have a correlate in the morphology of the dendritic tree, the main site of excitatory input onto PV^+^ neurons (Gulyás et al., 1999; Kameda et al., 2012). Quantitative analysis of the PV^+^ neuron dendrites is presented in Figure 5. The dendritic trees of individual L3-6 BCs, L2 BCs and L2 ChCs were superimposed and aligned, in the *y*-axis, relative to the pial surface and centered, in the *x*-axis, by the location of the soma (Fig. 5*A*). Polar histograms of distribution of the dendritic length relative to the cell body showed that L3-6 BC dendrites extend in an approximately multipolar fashion, and in a manner largely confined to the deep cortical layers (Fig. 5*B*). The dendrites of L2 BCs also extended in a multipolar manner, distributing branches mainly in layers 2 and 1 (Fig. 5*B*). In contrast, L2 ChC dendrites mostly extended above the cell soma into layer 1 (Fig. 5*B*), as in somatosensory cortex (Woodruff et al., 2011). Whereas the total dendrite length (mean ± SEM) did not differ significantly between PV^+^ neuron subtypes (L3-6 BCs: 3374 ± 517 µm, *n* = 7; L2 BCs: 2439 ± 262 µm, *n* = 5; L2 ChCs: 2016 ± 453 µm, *n* = 5; *F*_(2,14)_ = 2.452, *p* = 0.122, one-way ANOVA, Shapiro–Wilk *p* = 0.3419), the distribution of dendrite length as a function of distance from the pial surface differed markedly between L3-6 BCs and L2 BCs or L2 ChCs (Fig. 5*C*). The dendrites of L2 ChCs were largely restricted to layer 1, and only 1 of 5 ChCs extended dendritic branches deeper than 300 µm from the pial surface (Fig. 5*C*). For ChCs, the percentage of total dendrite length located at a distance ≤300 µm from the pial surface was 98.6 ± 1.4% (mean ± SEM, *n* = 5, range: 93–100%). Compared to ChCs, L2 BCs had dendrites more evenly distributed above and below the cell soma (Fig. 5*B*), yet L2 BC dendrites were largely confined to layer 1 and superficial layer 2, since 93.2 ± 3.8% of the L2 BC dendritic tree (mean ± SEM, *n* = 5, range: 78–100%) was found at a distance ≤300 µm from the pial surface. In contrast, only 2.1 ± 1.0% (mean ± SEM, *n* = 7, range: 0–8%) of the L3-6 BC dendrites was found at distances ≤300 µm from the pia, a significantly smaller percentage (χ^2^ = 12.655, *p* = 0.00179, Kruskal–Wallis ANOVA). These data therefore show that L3-6 BC dendrites integrate excitatory inputs at laminar locations that are largely non-overlapping with those of either L2 BC or L2 ChC dendrites, suggesting a morphologic correlate of the different carbachol effects on excitatory input. On the other hand, quantitative analysis showed that the axons of L3-6 BCs (Fig. 5*D*), projected into both deep and superficial layers in a multipolar fashion, as observed for L2 BC dendrites, albeit with greater projection length (Fig. 5*E*). Across cortical layers, the L3-6 BC axons projected in an approximately bimodal manner, with a tendency for a denser projection to the superficial layers (Fig. 5*F*), although the peak axonal length did not differ significantly (*t* = 0.355, *p* = 0.728, *n* = 7; Shapiro–Wilk, raw data *p* = 0.00127, log data *p* = 0.9959) between superficial layers (peak length, mean ± SEM: 383 ± 183 µm) and deep layers (peak length, mean ± SEM: 212 ± 82 µm). The percentage of L3-6 BC axonal arbor length found at a distance ≤300 µm from the pial surface was small (mean ± SEM, 1.7 ± 0.4%, range: 0–6.25%), suggesting that the laminar distribution of the L3-6 BC axons is similar to that of L3-6 BC dendrites. Hence, these data show that L3-6 BCs with carbachol-stimulated excitatory drive project axonal output mainly within layers 3-6, with weak output onto layer 2.

**Figure 6.**
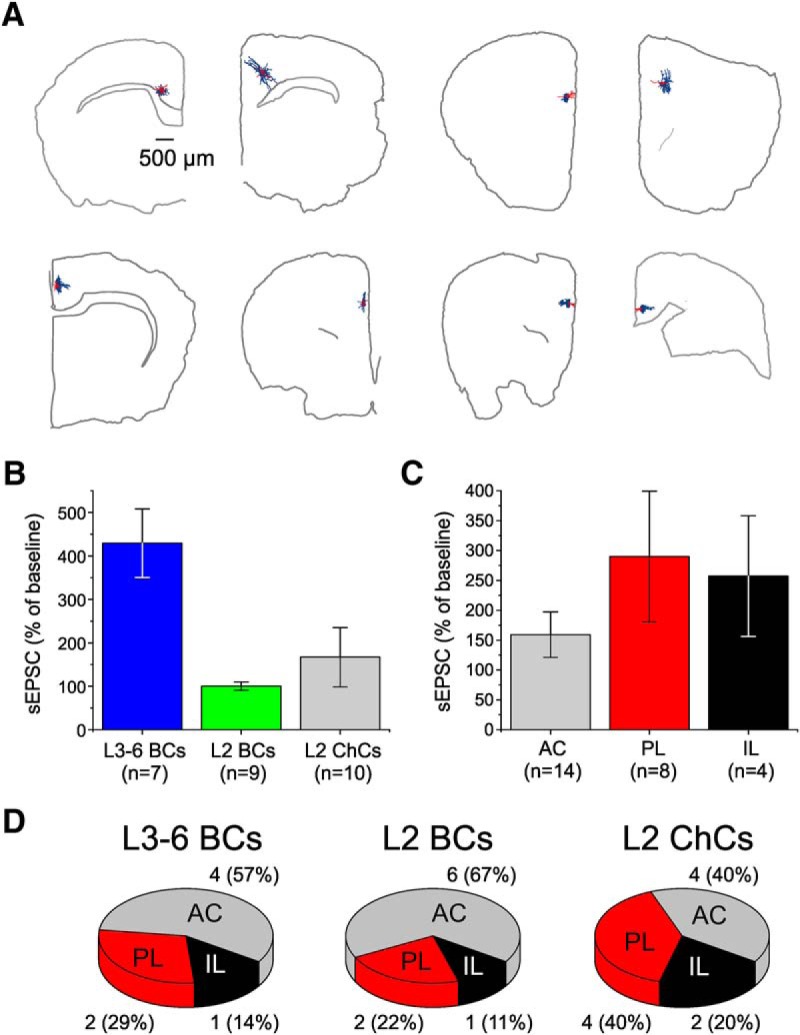
Distribution of the PV^+^ neurons tested for the effect of carbachol on sEPSCs, across cytoarchitectonic areas of the mouse PFC. ***A***, Examples illustrating the location of recorded PV^+^ neurons in different PFC slices. Shown are PV^+^ neurons for which we reconstructed the dendritic tree (red) and axonal arbors (blue), with the contour of each brain slice, and some landmarks indicating location of white matter tracks. Neurons were localized to the IL, PL, or AC cortex areas using the Allen Institute Mouse Brain Atlas (http://mouse.brain-map.org/static/atlas). ***B***, Potentiation by carbachol of the sEPSCs recorded from L3-6 BCs, L2 BCs and L2 ChCs. Data are from the experiments reported in Figures 2–4 after each neuron was located in the IL, PL, or AC areas. ***C***, Potentiation by carbachol of the sEPSCs recorded from PV^+^ neurons located in the AC, PL, or IL cortices. Two-way ANOVA of the data in parts ***B***, ***C*** performed using cell type and cytoarchitectonic area as the main factors showed a significant effect of cell type (*F*_(2,17)_ = 7.647, *p* = 0.00428), whereas there was no significant effect of cytoarchitectonic area (*F*_(2,17)_ = 1.233, *p* = 0.316) and no significant interaction (*F*_(4, 17)_ = 0.099, *p* = 0.981). ***D***, Charts illustrating the distribution of recorded L3-6 BCs, L2 BCs, and L2 ChCs across the three cytoarchitectonic areas of the mouse medial PFC.

The PV^+^ neurons from which we recorded sEPSCs were located throughout the IL, PL, and AC cytoarchitectonic areas of the mouse medial PFC. Thus, one possibility is that the different effect of carbachol on sEPSCs recorded from L3-6 BCs versus L2 BCs or L2 ChCs was due to a different areal localization of the L3-6 BCs versus the other PV^+^ neuron subtypes. To examine this idea, we determined the location by cytoarchitectonic area of the PV^+^ neurons tested with carbachol (Fig. 6*A*). For each PV^+^ cell we estimated the sEPSC frequency during the effects of carbachol as a percentage of the baseline value. Next, to compare the effect of carbachol on sEPSCs, we performed two-way ANOVA with cell type and cytoarchitectonic area as main factors (Fig. 6*B*). We found that cell type had a significant effect (*F*_(2,17)_ = 7.647, *p* = 0.00428), driven by the marked sEPSC potentiation in L3-6 BCs (Fig. 6*B*). However, the effect of cytoarchitectonic area (Fig. 6*C*) was not significant (*F*_(2,17)_ = 1.101, *p* = 0.355) and there was no significant interaction (*F*_(4,17)_ = 0.191, *p* = 0.939). These data suggest that, compared with L2 BCs or L2 ChCs, the L3-6 BCs with sEPSC potentiation by carbachol were not differentially localized by cytoarchitectonic area. An analysis of the anatomic distribution of the recorded neurons showed that most cells were localized in the AC cortex, and a minority of cells were in the IL cortex (Fig. 6*D*). However, we found no clear evidence of a bias in the area distribution of L3-6 BCs relative to L2 BCs or L2 ChCs (Fig. 6*D*). Because our anatomic analysis was limited by the small sample of neurons, we cannot rule out that in a larger sample we may detect significant differences in the effect of carbachol between cytoarchitectonic areas. However, since carbachol had a robust effect on sEPSCs on L3-6 BCs relative to L2 BCs and L2 ChCs that was independent of areal localization, our data indicate that the effect of cell type is stronger than any potential differences between PFC areas.

**Figure 7.**
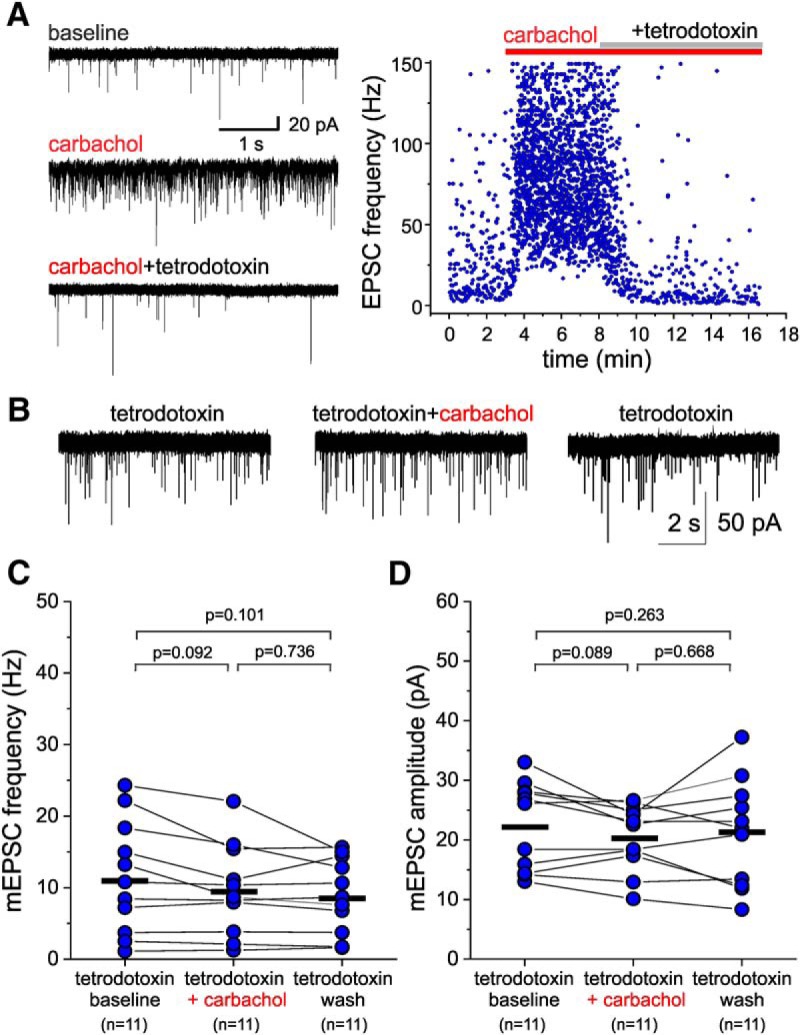
The voltage-dependent Na^+^ channel blocker tetrodotoxin prevents the carbachol-induced potentiation of sEPSCs recorded from L3-6 PV^+^ BCs. ***A***, left panel, sEPSCs recorded from a L3-6 BC during baseline, after addition of carbachol (20 µM) and after addition of tetrodotoxin (1 µM). Right panel, Time-course plot illustrating the effects of carbachol and tetrodotoxin addition on the instantaneous EPSC frequency (estimated as the inverse of the inter-event interval) for the EPSCs in the experiment in the left panel. ***B***, mEPSCs recorded in the continuous presence of tetrodotoxin (1 µM) before and after the addition of 20 µM carbachol. ***C***, Carbachol applied in the continuous presence of tetrodotoxin did not increase the mEPSC frequency, while producing a small but significant decrease when estimated 20 s before carbachol application (tetrodotoxin baseline), 20 s before beginning of washout (tetrodotoxin + carbachol), and after ≥10 min of washout (tetrodotoxin wash; one-way RM ANOVA *F*_(2,16)_ = 4.634, *p* = 0.022; shown in the figure are the *p* value results of Sidak-corrected, *post hoc* pairwise comparisons). The black horizontal bars indicate the mean value for each sample. ***D***, Carbachol applied in the continuous presence of tetrodotoxin did not have significant effects on mEPSC amplitude when estimated 20 s before carbachol application (tetrodotoxin baseline), 20 s before beginning of washout (tetrodotoxin + carbachol), and after ≥10 min of washout (tetrodotoxin wash; one-way RM ANOVA *F*_(2,16)_ = 1.281, *p* = 0.299; shown in the figure are the *p* value results of Sidak-corrected, *post hoc* pairwise comparisons). The black horizontal bars indicate the mean value for each sample.

The increase in excitatory drive in L3-6 BCs may reflect AChR-mediated stimulation of PN firing, since previous studies showed that carbachol stimulates PN activity. In this case, the effects of carbachol on L3-6 BC sEPSCs should be prevented by the voltage-dependent Na^+^ channel blocker tetrodotoxin, which inhibits action potential firing. Alternatively, it is possible that carbachol increases the probability of action potential-independent glutamate release at excitatory synapses onto L3-6 BCs, an effect that would be observed after tetrodotoxin application. To distinguish between these two possibilities, we tested the effects of carbachol (20 µM) applied in the presence of tetrodotoxin (1 µM). We found that the carbachol-induced increase in sEPSC frequency and amplitude in L3-6 BCs was reversed by the addition of tetrodotoxin (Fig. 7*A*). Moreover, when carbachol was applied in the continuous presence of tetrodotoxin (Fig. 7*B*), neither the miniature EPSC frequency (Fig. 7*C*) nor amplitude (Fig. 7*D*), showed significant increases by carbachol (one-way RM ANOVA, mEPSC frequency: *F*_(2,20)_ = 4.634, *p* = 0.022; mEPSC amplitude: *F*_(2,20)_ = 1.281, *p* = 0.299). These data are consistent with the idea that the effects of carbachol on sEPSCs involve stimulation of PN firing.

**Figure 8.**
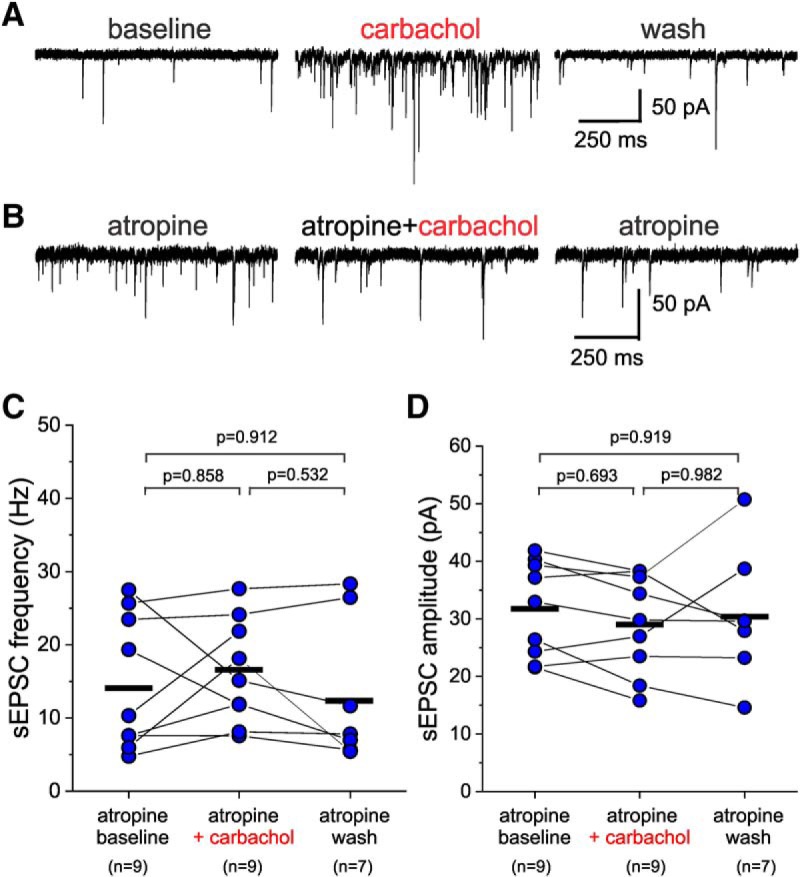
The non-selective mAChR antagonist atropine prevents the effects of carbachol on sEPSCs recorded from L3-6 PV^+^ BCs. ***A***, Examples of sEPSCs recorded in the continuous presence of the vehicle ethanol (0.1% V/V) from a layer 5 BC before (atropine baseline), during application of 20 µM carbachol (carbachol), and after washout (atropine wash). ***B***, Examples of sEPSCs recorded from a layer 5 BC in the continuous presence of the vehicle ethanol (0.1% V/V) and atropine (10 µM) before (baseline), during application of 20 µM carbachol (atropine+carbachol), and after washout (atropine wash). ***C***, Carbachol applied in the presence of atropine did not have significant effects on sEPSC frequency when estimated 20 s before carbachol application (atropine baseline), 20 s before beginning of washout (atropine+carbachol), and after ≥10 min of washout (atropine wash; one-way RM mixed model ANOVA, *F*_(2,16.46)_ = 0.813, *p* = 0.461; shown in figure are the results of Sidak-corrected, *post hoc* pairwise comparisons). The black horizontal bars indicate the mean value for each sample. ***D***, Carbachol applied in the presence of atropine did not have significant effects on sEPSC amplitude when estimated 20 s before carbachol application (atropine baseline), 20 s before beginning of washout (atropine+carbachol), and after ≥10 min of washout (atropine wash; one-way RM mixed model ANOVA, *F*_(2,16.11)_ = 0.524, *p* = 0.602; shown in figure are the results of Sidak-corrected, *post hoc* pairwise comparisons). The black horizontal bars indicate the mean value for each sample.

**Figure 9.**
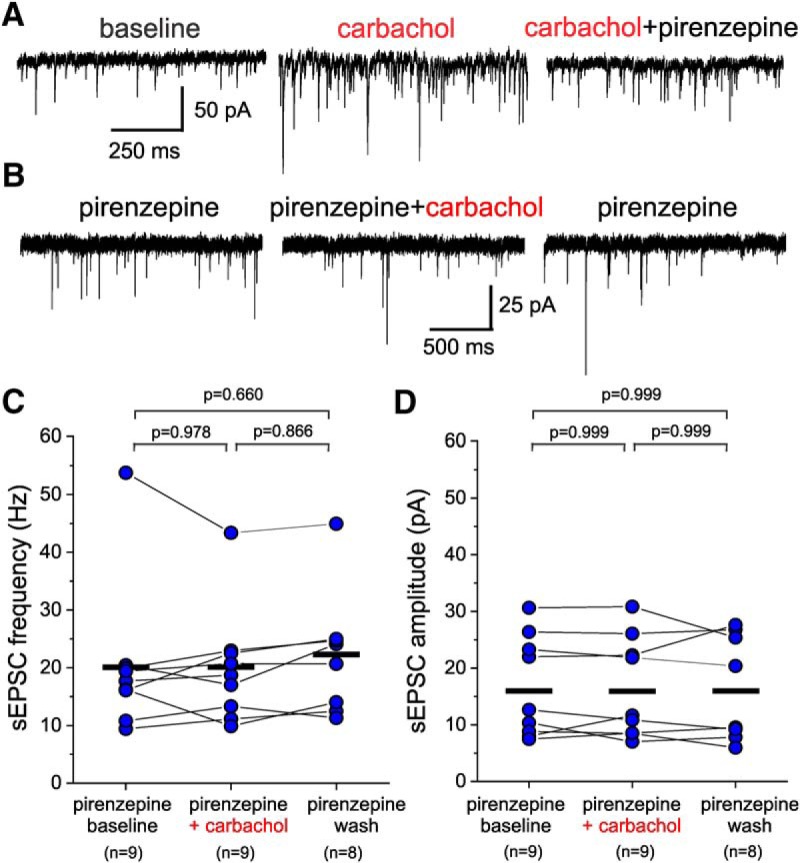
The M1 subtype-selective mAChR antagonist pirenzepine prevents the effects of carbachol on sEPSCs recorded from L3-6 PV^+^ BCs. ***A***, Examples of sEPSCs recorded at baseline (baseline), during application of 20 µM carbachol (carbachol), and after addition of 1 µM pirenzepine (carbachol+pirenzepine). ***B***, Examples of sEPSCs recorded from a layer 3-6 BC in the continuous presence of pirenzepine (pirenzepine), after addition of carbachol (carbachol+pirenzepine), and after carbachol washout (pirenzepine). ***C***, Carbachol applied in the presence of pirenzepine did not have significant effects on sEPSC frequency when estimated 20 s before carbachol application (pirenzepine baseline), 20 s before beginning of washout (pirenzepine + carbachol), and after ≥10 min of carbachol washout (pirenzepine wash; one-way RM ANOVA mixed model, *F*_(2,27.3)_ = 0.517, *p* = 0.575; shown in figure are the results of Sidak-corrected, *post hoc* pairwise comparisons). The black horizontal bars indicate the mean value for each sample. ***D***, Carbachol applied in the presence of pirenzepine did not have significant effects on sEPSC amplitude when estimated 20 s before carbachol application (pirenzepine baseline), 20 s before beginning of washout (pirenzepine + carbachol), and after ≥10 min of carbachol washout (pirenzepine wash; one-way RM ANOVA mixed model, *F*_(2,27.4)_ = 0.009, *p* = 0.991; shown in figure are the results of Sidak-corrected, *post hoc* pairwise comparisons). The black horizontal bars indicate the mean value for each sample.

In deep cortical layers, PNs express muscarinic and nicotinic AChRs (Obermayer et al., 2017; Radnikow and Feldmeyer, 2018), both of which can be activated by carbachol. However, carbachol stimulation of PN firing in PFC is dependent on mAChR activation (Gulledge et al., 2007; Gulledge et al., 2009; Pafundo et al., 2013). Thus, to determine whether the increase in sEPSC frequency and amplitude by carbachol is consistent with mAChR stimulation of PN firing, we examined the effects of carbachol on sEPSCs recorded from L3-6 BCs in the presence of the mAChR antagonist atropine. We first tested whether the effects of carbachol are modified by the presence of ethanol, which we used as the vehicle for atropine. We found that in the continuous presence of ethanol at vehicle concentrations (0.1% v/v), carbachol produced a marked increase in the sEPSC frequency and amplitude in deep layer BCs, comparable to that observed in the absence of ethanol (Fig. 8*A*). In contrast, in the presence of atropine (10 µM), carbachol (20 µM) application did not alter the sEPSCs recorded from L3-6 BCs (Fig. 8*B*), and neither sEPSC frequency (Fig. 8*C*) nor sEPSC amplitude (Fig. 8*D*) were changed significantly (one-way RM mixed model ANOVA, sEPSC frequency: *F*_(2,16.46)_ = 0.813, *p* = 0.461, sEPSC amplitude: *F*_(2,16.11)_ = 0.524, *p* = 0.602). We additionally used the M1-selective mAChR antagonist pirenzepine (Gulledge and Stuart, 2005; Carr and Surmeier, 2007), since in previous studies the modulation of PFC PN firing by mAChR activation was abolished by M1 subtype mAChR antagonists or genetic ablation of the M1 mAChR (Gulledge and Stuart, 2005; Carr and Surmeier, 2007; Gulledge et al., 2007, 2009). The carbachol (20 µM)-induced increase in sEPSC frequency and amplitude in L3-6 BCs was reversed by the addition of pirenzepine (1 µM; Fig. 9*A*). Moreover, when carbachol (20 µM) was applied in the continuous presence of 1 µM pirenzepine (Fig. 9*B*), the increases in both sEPSC frequency (Fig. 9*C*) and sEPSC amplitude (Fig. 9*D*) were abolished [sEPSC frequency: *F*_(2,27.3)_ = 0.565, *p* = 0.575, one-way RM mixed model ANOVA (on ranks); sEPSC amplitude: *F*_(2,27.4)_ = 0.009, *p* = 0.991, one-way RM mixed model ANOVA (on ranks)]. These data show that the effects of carbachol on sEPSCs recorded from L3-6 BCs are mediated by M1 mAChRs.

**Figure 10.**
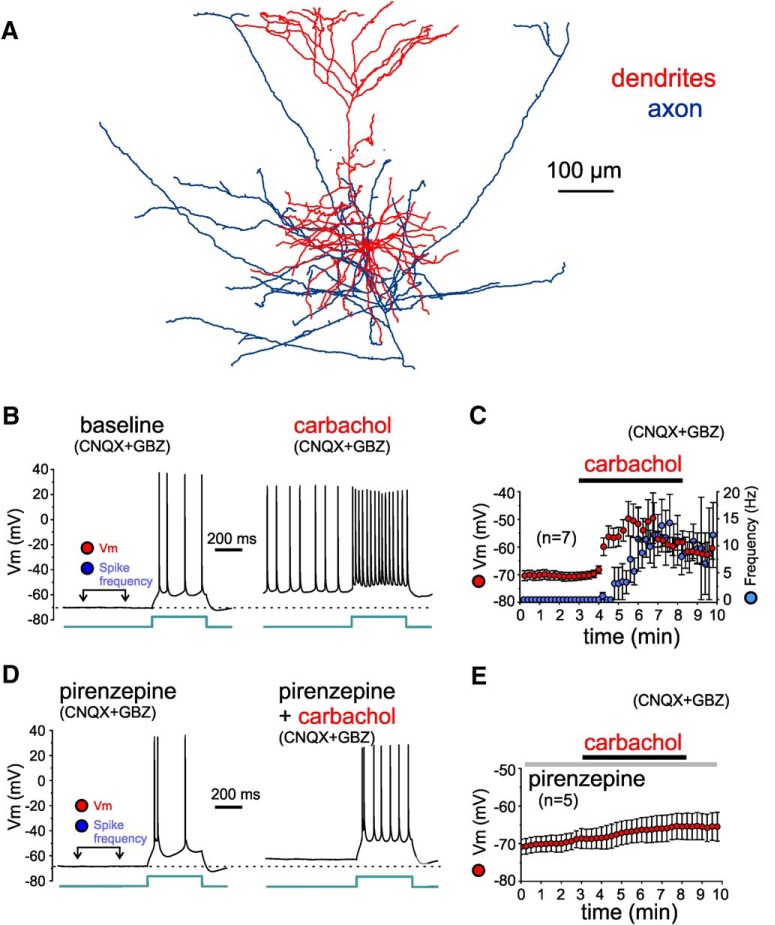
Effects of carbachol on the membrane potential and excitability of L3-6 PNs, tested in the presence of synaptic receptor blockers (CNQX and gabazine). ***A***, Reconstruction of the dendritic tree of a layer 5 PN for which the effects of carbachol were assessed during current clamp recordings. ***B***, Examples of membrane potential (Vm) recorded from a layer 5 PN before (baseline) and during application of 20 µM carbachol (carbachol), in the presence of synaptic receptor blockers CNQX and gabazine (GBZ). The time window for measurements of Vm (minimum value within the window) and of spike frequency (independent of injection of current steps) is illustrated in the baseline recording example. The injected current step had identical amplitude (70 pA) in baseline and carbachol conditions. Note that during the depolarization induced by carbachol, action potential amplitude was typically reduced. ***C***, Plot illustrating the time course of changes in Vm (red) and in spike frequency (blue) measured during the time window indicated in ***B***. Carbachol depolarized the membrane potential significantly by 2 min of carbachol application (baseline: –71 ± 2 mV, carbachol: –52 ± 5 mV, *n* = 7, *t*_(6)_ = 5.04, *p* = 0.0023, paired *t* test). Note that for several PNs the strong depolarization by carbachol produced depolarization block of action potential firing, which was prevented by hyperpolarizing current injection (see text in Results). Measurements obtained during hyperpolarizing current injection were not included in this time-course plot. ***D***, Examples of Vm recordings from a layer 5 PN in the continuous presence of the M1 mAChR antagonist pirenzepine (1 µM) before (pirenzepine) and during application of 20 µM carbachol (pirenzepine+carbachol). Vm and spike frequency measurements were performed as indicated in ***C***. The injected current step had identical amplitude (80 pA) in baseline and carbachol conditions. Note that in the presence of pirenzepine, the carbachol-induced depolarization was subthreshold and did not evoke spikes independent of the injected current steps. ***E***, Plot illustrating the time course of changes in Vm. In the presence of pirenzepine, carbachol depolarized the membrane potential, but the depolarization was small and remained below action potential threshold. The depolarization was not significant by 2 min of application but peaked at significant levels by 5 min (baseline: –69 ± 2 mV, 2 min pirenzepine+carbachol: –67 ± 3 mV. 5 min pirenzepine+carbachol: –65 ± 3 mV, *n* = 5, *F*_(2,8)_ = 8.555, *p* = 0.0103, one-way RM ANOVA; baseline vs 2 min: *p* = 0.239, baseline vs 5 min: *p* = 0.0034, Dunnett's *post hoc* tests).

Our findings that carbachol increases the excitatory drive on L3-6 BCs suggest that carbachol initiates action potential firing in PNs, some of which are a source of excitatory synaptic input onto the L3-6 BCs. Previous findings suggest that mAChR-mediated stimulation of action potential firing by carbachol is more pronounced in PNs from deep layers (Dembrow et al., 2010; Joshi et al., 2016; Baker et al., 2018). Our data in Figure 5 also show that the dendrites of L3-6 BCs integrate excitatory input mainly in deep layers, in contrast with the dendrites of L2 PV^+^ cells, which receive input in layers 1 and 2. Thus, we obtained current clamp recordings from PNs in deep layer 3 and layer 5 (L3-5 PNs; Fig. 10*A*), in the continuous presence of glutamate and GABA_A_ receptor antagonists, to assess if carbachol initiates action potential firing in L3-5 PNs. We additionally applied depolarizing current steps to evoke spiking, assessing changes in the response to excitatory currents. The response to depolarizing current steps during baseline before carbachol application showed that the recorded L3-5 PNs (*n* = 7) had either regular spiking (*n* = 5) or bursting properties (*n* = 2) typical of PNs (Fig. 10*B*,*D*). After monitoring the resting membrane potential and the response to depolarizing current steps (Fig. 10*B*) for at least 3 min of baseline recordings, we bath-applied carbachol (20 µM) for 5 min. Carbachol depolarized the L3-5 PN membrane potential, reaching action potential threshold and eliciting firing independent of current injection in 6 of 7 recorded L3-5 PNs (Fig. 10*B*). As expected, the membrane depolarization enhanced the response to the depolarizing current steps (Fig. 10*B*), and this effect was reversed or partly reversed by injection of constant hyperpolarizing current (data not shown). By 2 min of carbachol application, the depolarization developed significant values (membrane potential, mean ± SEM, baseline: –71 ± 2 mV, carbachol: –52 ± 5 mV, *n* = 7, *t*_(6)_ = 5.04, *p* = 0.0023, paired sample Student’s *t* test), and rapidly reached suprathreshold levels. Figure 10*C* shows the time course of the carbachol-induced PN depolarization and the frequency of spikes evoked by this depolarization, both measured in a time window just before the injected current steps, as indicated in Figure 10*B*. The carbachol-induced depolarization directly produced action potentials at a frequency of up to 15 Hz independent of the response to the injected current steps (Fig. 10*C*). The depolarization and action potential firing evoked by carbachol were reversed with drug washout in two of seven L3-5 PNs, but in most cells, the carbachol effects were persistent and did not reverse completely after up to 20 min of wash (data not shown). In four of seven recorded L3-5 PNs, within 2 min of eliciting spiking carbachol produced depolarization block of the action potentials, which was readily reversed by hyperpolarizing current injection (data not shown).

We next tested whether the M1-selective mAChR antagonist pirenzepine reduced the action potential firing elicited in L3-5 PNs by carbachol, as expected if the effect of carbachol contributes to the increase of excitatory drive onto L3-6 BCs (Figs. 2, 9). In recordings from L3-5 PNs (*n* = 5, regular spiking *n* = 4, bursting *n* = 1), in the presence of pirenzepine (1 µM), carbachol (20 µM) did not depolarize the cells or produced a small depolarization that did not directly elicit action potential firing in any of the five L3-5 PNs tested (Fig. 10*D*). By 2 min of carbachol application in the presence of pirenzepine, the depolarization was not significant (Fig. 10*E*), but it peaked at significant levels by 5 min (membrane potential, mean ± SEM, pirenzepine baseline: –69 ± 2 mV, 2-min pirenzepine+carbachol: –67 ± 3 mV, 5-min pirenzepine+carbachol: –65 ± 3 mV. *n* = 5, *F*_(2,8)_ = 8.555, *p* = 0.0103, one-way RM ANOVA, Sidak *post hoc* tests, baseline vs 2 min: *p* = 0.372, baseline vs 5 min: *p* = 0.010). In the presence of pirenzepine, carbachol did not initiate PN spikes independent of the injected current steps, therefore these experiments did not produce spike frequency measures (Fig. 10*E*). Our current clamp recordings thus showed that carbachol produces a suprathreshold depolarization that elicits L3-5 PN firing via activation of M1 mAChRs.

**Figure 11.**
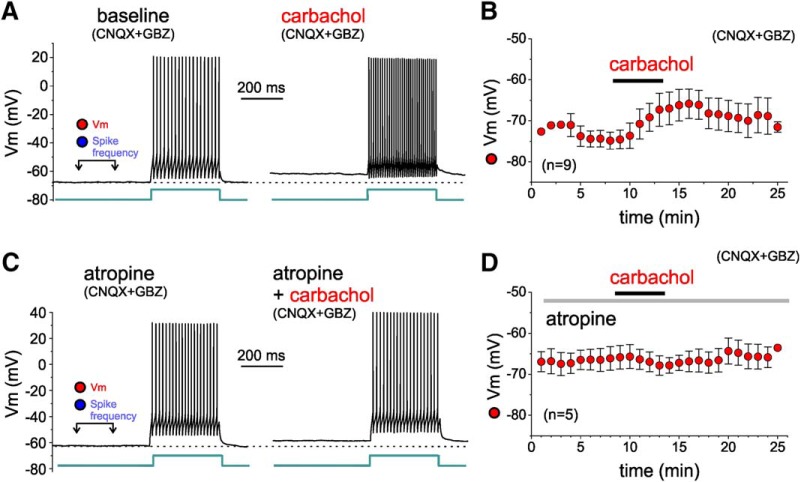
Effects of carbachol on the membrane potential and excitability of L3-6 BCs, tested in the presence of synaptic receptor blockers (CNQX and gabazine). ***A***, Examples of membrane potential (Vm) recorded from a layer 5 BC before (baseline) and during application of 20 µM carbachol (carbachol), in the presence of synaptic receptor blockers CNQX and gabazine (GBZ). Measurements of Vm and of spike frequency were obtained as in Figure 10. The injected current step had identical amplitude (80 pA) in baseline and carbachol conditions. Note that carbachol application did not elicit action potential firing. ***B***, Plot illustrating the time course of changes in Vm measured during the time window indicated in ***A***. Carbachol depolarized the membrane potential significantly by 5 min of application and peaked at 8 min [baseline: 74 ± 2 mV, carbachol 5 min: –69 ± 3 mV, carbachol 8 min: –66 ± 4 mV, *n* = 9; one-way RM ANOVA (on ranks), *F*_(1.12,16)_ = 4.490, *p* = 0.060, baseline vs 5 min: *p* = 0.024, baseline vs 8 min: *p* = 0.170, Sidak-corrected, *post hoc* pairwise comparisons]. ***C***, Examples of Vm recordings from a layer 5 BC in the continuous presence of the mAChR antagonist atropine (10 µM) before (atropine) and during application of 20 µM carbachol (carbachol). Vm measurements were performed as indicated in ***C***. The injected current step had identical amplitude (110 pA) in atropine and carbachol conditions. ***D***, Plot illustrating the time course of changes in Vm. In the presence of atropine, the depolarization of the L3-6 BC membrane potential was abolished (atropine baseline: –66 ± 3 mV, atropine+carbachol 5 min: –67 ± 2 mV, atropine + carbachol 8 min: –67 ± 4 mV, *n* = 5; one-way RM ANOVA, on ranks, *F*_(2,8)_ = 1.500, *p* = 0.285).

mAChR expression is significant in PNs and also in PV^+^ neurons (Paul et al., 2017; Oda et al., 2018), and mAChR stimulation increases the intrinsic excitability of PV^+^ neurons in PFC (Pafundo et al., 2013; Yi et al., 2014). Hence, our data showing increased excitatory input onto PV^+^ L3-6 BCs, and previous findings of increased L3-6 BC intrinsic excitability, suggest that mAChR activation has overall excitatory effects on PV^+^ BCs in layers 3-6. However, previous studies assessing the effects of AChR stimulation on the membrane potential of PV^+^ neurons from the hippocampus or from frontal, somatosensory, or visual cortices reported conflicting results, including a membrane potential hyperpolarization that would have inhibitory effects (Kawaguchi, 1997; Xiang et al., 1998; Yi et al., 2014; Bell et al., 2015; Yi et al., 2015; Wenger Combremont et al., 2016). To determine the effects of carbachol on the membrane potential and intrinsic excitability of PV^+^ neurons in PFC, we obtained current clamp recordings from L3-6 PV^+^ neurons in the continuous presence of glutamate and GABA_A_ receptor antagonists. All recorded neurons exhibited fast spiking properties consistent with those of PV^+^ cells (Fig. 11*A*,*C*) and had BC morphology (data not shown). Figure 11*A* shows that carbachol (20 µM) applied for 5 min depolarized the membrane potential of the L3-6 BCs (Fig. 11*B*). The carbachol-induced depolarization was small (mean ± SEM, baseline: –74 ± 2 mV, carbachol 5 min: –69 ± 3 mV), but was significant relative to baseline (paired sample Student’s *t* test, *t*_(8)_ = 2.565, *p* = 0.0333). Contrasting with the strong suprathreshold depolarization of L3-5 PNs, the L3-6 BC depolarization was largely subthreshold, since in most L3-6 BCs (eight of nine) carbachol did not directly elicit firing (Fig. 11*A*). As expected, the membrane depolarization enhanced the response to depolarizing current steps (Fig. 11*A*) in all L3-6 BCs, and this effect was reversed or partly reversed by injection of constant hyperpolarizing current (data not shown). However, carbachol did not initiate PV^+^ neuron spikes independent of the injected current steps in most L3-6 BCs (eight of nine), and thus we did not obtain measures of spike frequency (Fig. 11*B*).

PV^+^ neurons express mAChRs of M1, M2 and M3 subtypes (Hajos et al., 1998; Paul et al., 2017; Oda et al., 2018), as well as nicotinic AChRs (Muñoz and Rudy, 2014). Thus, to assess whether the depolarization of PV^+^ cells is mediated by mAChRs, we applied carbachol (20 µM) in the continuous presence of the non-selective mAChR antagonist atropine (10 µM), in addition to glutamate and GABA_A_ receptor antagonists (Fig. 11*C*). As shown in Figure 11*D*, the L3-6 BC depolarization was abolished in the presence of atropine (membrane potential, mean ± SEM, atropine baseline: –66 ± 3 mV, atropine+carbachol 5 min: –67 ± 2 mV, atropine + carbachol 8 min: –67 ± 4 mV, *n* = 5; one-way RM ANOVA, on ranks, *F*_(2,8)_ = 1.500, *p* = 0.285). In the presence of atropine, carbachol did not initiate PV^+^ neuron spikes independent of the injected current steps; therefore, we could not obtain measures of spike frequency in this condition (Fig. 11*D*). These data show that, via mAChRs, carbachol produces a depolarization of the L3-6 BC membrane potential which, relative to the depolarization of L3-6 PNs, is small and does not initiate action potential firing. Given that CNQX, the antagonist used here to block glutamate synaptic transmission does not inhibit NMDA or metabotropic glutamate receptors, we cannot rule out that these receptors mediate the observed subthreshold depolarization (for additional details, see Discussion).

**Figure 12.**
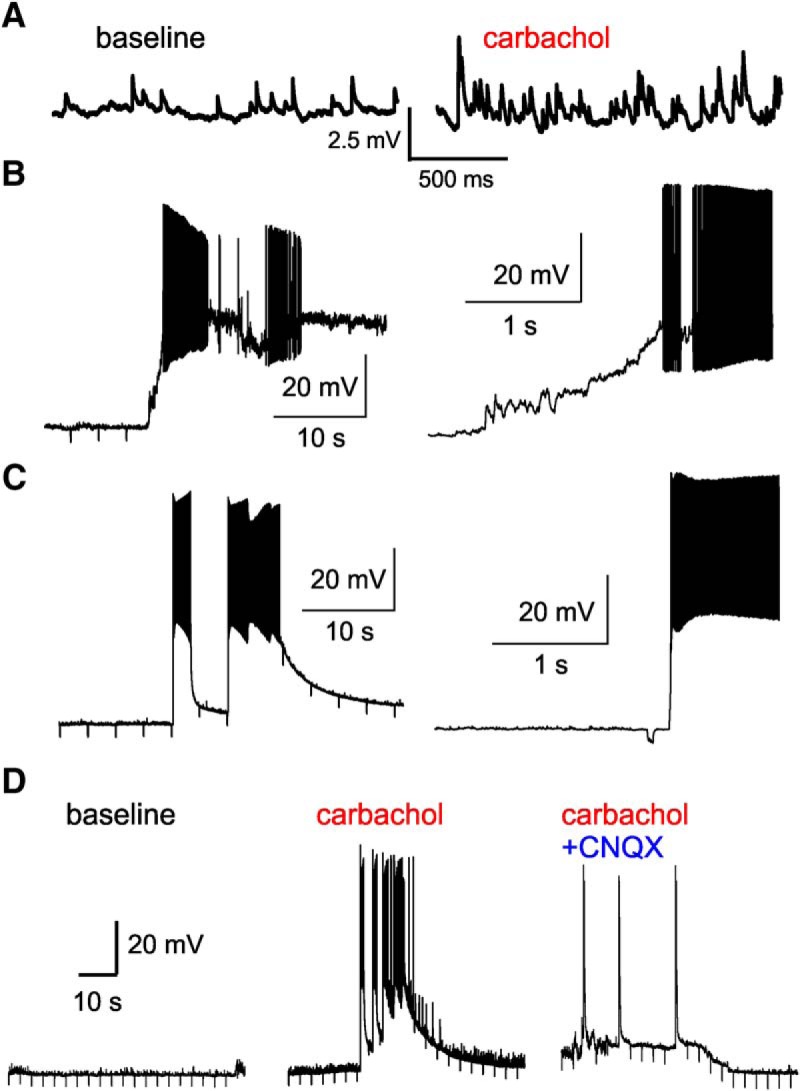
Action potential bursts induced by carbachol in PV^+^ L3-6 BCs in the absence of CNQX. ***A***, Examples of sEPSPs recorded from a BC during baseline and after addition of carbachol (20 µM) to the bath solution. ***B***, left, Example of an action potential burst elicited in a L3-6 BC by carbachol, which was preceded by a depolarization ramp with a simultaneous increase in sEPSP activity. Right, An expanded view of the burst illustrated in the left panel, showing the development of a depolarizing plateau, which was associated with inactivation of the Na^+^ spike mechanisms. The relatively brief downward deflections in the membrane potential here and in ***C***, ***D*** are the responses to hyperpolarizing current steps used to monitor recording conditions, as well as the cells input resistance and time constant. ***C***, left panel, Example of an action potential burst evoked by carbachol that was not preceded by a depolarizing ramp. Right panel, An expanded view of the left panel showing that this burst was not associated with spike inactivation and depolarization plateau, but showed a decay in the membrane potential before a subsequent burst was elicited in the presence of carbachol. ***D***, Example traces showing recordings from a PV^+^ L3-6 BC during the baseline period (left panel), after addition of carbachol (20 µM, middle panel), and after addition of CNQX (10 µM, right panel). Note that shortly after the addition of CNQX, bursting activity, the plateau depolarization, and the sEPSPs were all abolished.

The results reported in Figure 11, and those of previous studies (Kawaguchi, 1997; Gulledge et al., 2007; Pafundo et al., 2013; Yi et al., 2014), suggest that mAChRs do not directly evoke action potential firing in PV^+^ cells. However, since carbachol increased the excitatory drive (Fig. 2) and produced a subthreshold depolarization (Fig. 11), these effects, combined, could recruit L3-6 BC activity. To test this idea, we performed current clamp recordings from L3-6 BCs with GABA_A_ receptor-mediated inhibition blocked, but glutamate receptor-mediated transmission intact. During 5 min of baseline recording, the L3-6 BCs displayed relatively frequent spontaneous EPSPs (sEPSPs) and stable resting membrane potential values (Fig. 12*A*). After carbachol (20 µM) was applied, most L3-6 BCs (12/13) displayed an increase in the frequency and amplitude of sEPSPs (Fig. 12*A*), consistent with the potentiation of sEPSCs by carbachol (Fig. 2). In the absence of CNQX, carbachol stimulated the firing of action potential bursts in 11/13 L3-6 BCs (Fig. 12*B*,*C*), thus having effects markedly different from those observed with excitatory input blocked. Most L3-6 BCs (7/11) were silent during baseline but displayed bursts of action potentials by 3.1 ± 0.7 min of carbachol application. Other cells (4/11) displayed bursts at low frequency during baseline, and the effects of carbachol increased the number of bursts observed. Some action potential bursts were preceded by a long depolarizing ramp simultaneous with a marked increase in the sEPSP number (Fig. 12*B*, right panel). Other firing bursts were not preceded by a depolarizing ramp or increase in sEPSPs but were initiated after a sharp depolarization (Fig. 12*C*, right panel). The bursts preceded by a depolarizing ramp were typically associated with a plateau depolarization that eventually inactivated the Na^+^ spikes (Fig. 12*B*, left panel). In contrast, spike bursts initiated by sharp depolarizations without a ramp usually ended when the membrane potential started a decay to baseline values (Fig. 12*C*, left panel). The depolarizing ramps may reflect direct input from excitatory cells that progressively increase their activity until driving a burst in the network, whereas episodes without a preceding ramp may reflect bursts propagating in the network after initiation at a distant site. The mechanisms producing these different modes of carbachol-induced bursts of spikes are unclear, but do not seem to depend on unique properties of each cell, because single PV^+^ cells alternated between these modes of burst spiking. Independent of the underlying mechanisms, all firing episodes, preceded by a depolarizing ramp or not, were dependent on glutamate synaptic transmission since they were eliminated by CNQX (Fig. 12*D*). These results show that the increase in excitatory synaptic drive by carbachol can evoke action potential firing in L3-6 BCs.

## Discussion

### mAChR stimulation increases the excitatory drive onto PV^+^ neurons in a cell type- and layer-specific manner

To gain insight into the cellular mechanisms by which cholinergic neuromodulation shapes network activity in the PFC, we assessed the effects of AChR activation on PV^+^ neurons. Previous studies of mouse PFC showed that AChR stimulation facilitates PN firing (Dembrow et al., 2010; Obermayer et al., 2017; Baker et al., 2018; Radnikow and Feldmeyer, 2018), an effect that may enhance the excitatory synaptic drive onto PV^+^ cells. Therefore, we characterized the effects of carbachol on excitatory drive by measuring changes in the frequency and amplitude of sEPSCs recorded from PV^+^ neurons. The effects of carbachol on PFC PNs are generally stronger in deeper layers (Obermayer et al., 2017; Radnikow and Feldmeyer, 2018). Moreover, most of these effects are mediated by stimulation of mAChRs, particularly of the M1 subtype (Gulledge et al., 2009; Dembrow et al., 2010; Baker et al., 2018). Thus, we predicted that carbachol would increase the excitatory drive onto PV^+^ neurons via M1 mAChR activation and have stronger effects in deep cortical layers, where carbachol is more likely to elicit PN firing. Our data are consistent with these predictions, since we found that carbachol increased the excitatory drive selectively in PV^+^ BCs of L3-6, and the effect was blocked by the broad spectrum mAChR antagonist atropine or by the M1-selective mAChR antagonist pirenzepine.

The sources of excitatory synaptic input onto PV^+^ neurons in PFC have not been well characterized; thus, it was not possible to predict whether carbachol would affect the excitatory drive differentially onto ChCs and BCs. In PV^+^ neurons from mouse PFC, EPSCs can originate from nearby PNs (Pafundo et al., 2013; Yang et al., 2014; Lu et al., 2017) or from long-range inputs arriving from the contralateral PFC, hippocampus, amygdala, or mediodorsal thalamus (McGarry and Carter, 2016; Bogart and O'Donnell, 2018). Importantly, some data indicate that transmission at long-range excitatory inputs is suppressed by mAChR activation (Picciotto et al., 2012), although ACh neuromodulation may also downregulate local excitatory synaptic input from PNs onto other PNs (Tsodyks and Markram, 1997) or onto PV^+^ cells (Levy et al., 2008). Thus, it seems likely that carbachol increases the excitatory drive onto PV^+^ neurons primarily by facilitating the firing of neighboring PNs located in the PFC brain slices. Consistent with this possibility, we found that carbachol produced a strong suprathreshold depolarization and readily evoked action potential firing in L3-5 PNs. In mouse PFC, PNs provide strong excitatory input to PV^+^ BCs, and, albeit with lower probability, onto a subpopulation of ChCs that are PV-negative (Taniguchi et al., 2013; Lu et al., 2017). Whether PV^+^ ChCs also receive significant input from local PNs remains unclear. The data reported here suggest that carbachol increases the excitatory drive onto PV^+^ neurons by facilitating the firing of a subset of PNs that innervate L3-6 PV^+^ BCs, but not L2 BCs or ChCs. The effects of carbachol on PNs are stronger in deeper PFC layers (Obermayer et al., 2017; Radnikow and Feldmeyer, 2018), and we found that carbachol stimulates the firing of PNs located in deep layers. Thus, it is likely that the increase in excitatory drive onto L3-6 BCs originated from deep layer PNs. The local axon collaterals from deep layer PNs project mostly within deep layers (Markram et al., 2015; Reimann et al., 2017), and their projections to layers 1 and 2 (Fig. 10*A*) are very limited (Markram et al., 2015; Reimann et al., 2017). Therefore, the laminar distribution of PV^+^ neuron dendrites reported here suggests that only L3-6 BCs have dendritic trees overlapping with the innervation fields from deep layer PNs. These morphologic properties are consistent with the idea that the increase in excitatory drive onto deep layer BCs originates from carbachol stimulation of PN firing in deep layers. As mentioned above, PNs provide input onto ChCs that are PV-negative and are mostly located in PFC layers 5 and 6 (Taniguchi et al., 2013). However, we did not study the PV-negative ChCs, since we recorded exclusively from PV^+^ neurons (genetically labeled with GFP), and all the GFP+ cells in layers 3-6 were BCs. Therefore, the effects of carbachol on the PV-negative subpopulation of deep layer ChCs remain to be determined.

Whereas carbachol, a carbamate analog of ACh resistant to hydrolysis by cholinesterases, is an agonist at both muscarinic and nicotinic AChRs, the stimulation by carbachol of PN firing in PFC is mediated by mAChRs (Gulledge et al., 2009; Obermayer et al., 2017; Baker et al., 2018; Radnikow and Feldmeyer, 2018). Moreover, in PFC, mAChRs also mediate the increase in PV^+^ neuron excitability by carbachol (Pafundo et al., 2013). Our finding that the increase in excitatory drive onto PV^+^ BCs in L3-6 was prevented by mAChR antagonists (atropine or pirenzepine) is therefore consistent with previous observations that many of the effects of carbachol in neocortex, and specifically in PFC, are mediated by mAChRs.

### Activation of mAChRs stimulates L3-5 PN firing directly and stimulates L3-6 BC firing indirectly, via glutamate synapses

Our findings that tetrodotoxin and pirenzepine block the carbachol-induced increase in excitatory drive onto L3-6 BCs are consistent with the idea that M1 mAChR activation stimulates firing of PNs that are a source of excitatory input onto L3-6 BCs. Here we found that M1 mAChR stimulation by carbachol elicits PN firing, as in previous studies (Gulledge and Stuart, 2005; Carr and Surmeier, 2007), and, moreover that most (∼86%) of the L3-5 PNs showed direct excitation by M1 mAChR activation. The large percentage observed here of PNs directly stimulated by carbachol, and previous studies showing that local PNs are a significant source of excitatory input onto PV^+^ BCs (Pafundo et al., 2013; Yang et al., 2014; Lu et al., 2017) support the idea that the increase in excitatory drive onto L3-6 BCs results from stimulating firing in nearby PNs. In a previous study of mouse PFC, carbachol evoked firing in a minority (∼30%) of the recorded PNs (Pafundo et al., 2013), contrasting with the ∼86% reported here. This discrepancy may be explained by the layer-dependent effects of cholinergic neuromodulation, since here we studied PNs in deep layers 3-5, versus mid/superficial layer 3 neurons in the previous study (Pafundo et al., 2013). Furthermore, here, but not in the previous study (Pafundo et al., 2013), we superfused the recording chamber at high flow rate, a condition that enhances the magnitude and stability of the effects of carbachol in brain slices (Hajos et al., 2009).

The increases in sEPSC frequency and amplitude in L3-6 BCs by carbachol seem to reflect the stimulation of PN firing, since they were abolished by application of tetrodotoxin. Because single presynaptic PN axons establish multiple synapses with each postsynaptic PV^+^ cell (Krimer and Goldman-Rakic, 2001; Wang et al., 2002), action potential firing in a PN simultaneously activates multiple synapses onto the PV^+^ cell, producing EPSCs larger than those resulting from release at single synapses. Thus, the increase in sEPSC amplitude observed here may be caused by an increased proportion of sEPSCs produced by simultaneous release from multiple synapses when carbachol stimulates PN firing. Importantly, in studies of synaptically-connected pairs (Levy et al., 2008; Pafundo et al., 2013) carbachol decreased the amplitude of the first response evoked in PV^+^ neurons by stimulus trains in nearby presynaptic PNs, while having small or no effect on subsequent responses that display short-term depression (Levy et al., 2008; Pafundo et al., 2013). Together, the data suggest that in addition to stimulating the firing of PNs presynaptic to PV^+^ cells, mAChR activation modulates presynaptic action potential-dependent glutamate release mechanisms, and in a manner that may optimize PN-to-PV^+^ cell synaptic transmission during repetitive PN firing (Levy et al., 2008; Pafundo et al., 2013).

Previous studies of PV^+^ neurons in mouse PFC suggested that AChR activation does not directly depolarize or hyperpolarize the membrane of PV^+^ neurons (Kawaguchi, 1997; Gulledge et al., 2007; Pafundo et al., 2013; Yi et al., 2014). In contrast, other studies reported varying effects of AChR stimulation on PV^+^ cells, including a hyperpolarizing effect that may inhibit PV^+^ neuron activity. Many of these effects were characterized in other cortical regions (Xiang et al., 1998; Yi et al., 2014; Bell et al., 2015; Yi et al., 2015; Wenger Combremont et al., 2016), and the AChR effects in PV^+^ neurons from PFC have been assessed in only a limited number of studies. We therefore tested the effects of carbachol on the membrane potential and excitability of PV^+^ neurons in mouse PFC with synaptic transmission blocked. We found that carbachol depolarized the L3-6 BCs via mAChR activation, but the depolarization was small and did not evoke action potential firing, in contrast to the direct suprathreshold effect observed in L3-5 PNs. To block excitatory synaptic transmission, we used the non-NMDA glutamate receptor antagonist CNQX. Thus, we cannot rule out that PN firing stimulated by carbachol increases glutamate release and activates NMDA or metabotropic glutamate receptors mediating the depolarization produced in the presence of CNQX. However, NMDA receptors are unlikely to mediate such depolarization in PV^+^ neurons because in these neurons NMDA receptor-mediated synaptic currents are very small relative to non-NMDA responses (Rotaru et al., 2011; Bogart and O'Donnell, 2018). Interestingly, PV^+^ neurons display a tonic NMDA receptor-mediated current that could contribute to the depolarization observed in the present experiments, although at negative membrane potentials such as those studied here, the tonic NMDA current is small (Povysheva and Johnson, 2012). We additionally tested the prediction that the enhanced excitatory drive, likely combined with the depolarization and increased excitability of PV^+^ neurons, may elicit L3-6 BC firing. We found that carbachol application with glutamate synaptic transmission intact evoked firing in 85% (11/13) of the L3-6 BCs. In contrast, only 11% (1/9) of the L3-6 BCs fired in response to carbachol application when glutamatergic transmission was blocked, demonstrating the capacity of the carbachol-induced excitatory drive to recruit L3-6 BC activity.

### Functional consequences of mAChR-mediated potentiation of excitatory drive onto PV^+^ BCs

PV^+^ neuron activation by excitatory synaptic input is crucial to generate γ oscillations in the pyramidal-interneuron-γ-network (PING) model (Fisahn et al., 1998; Whittington et al., 2000). In the PING model, which is supported by empirical data (Hajos and Paulsen, 2009; Sohal et al., 2009; Buzsáki and Wang, 2012; Neske et al., 2015; Salkoff et al., 2015), PV^+^ neuron recruitment by synaptic input from nearby PNs generates the rhythmic feedback inhibition that synchronizes the PN network (Whittington et al., 2000; Börgers and Kopell, 2003; Borgers, 2017). γ Oscillations are prominent in mouse PFC (Ruiz-Mejias et al., 2011; Cho et al., 2015; Howe et al., 2017; Cao et al., 2018), seem to preferentially involve BCs relative to ChCs (Gulyás et al., 2010; Dugladze et al., 2012; Massi et al., 2012), and depend on mAChRs (Rodriguez et al., 2004, 2010; Janiesch et al., 2011; Howe et al., 2017). Therefore, our current data suggest the prediction, to be tested in future studies, that the enhancement of excitatory input onto PV^+^ BCs is a mechanism by which mAChR activation contributes to γ oscillation production.

γ Band synchrony is thought to be crucial for transmission of information between cortical areas (Fries, 2015). Thus, if the enhanced excitatory input onto PV^+^ BCs contributes to γ oscillation production, this could be an important process by which cholinergic neuromodulation supports cognitive function. Consistent with a crucial role of ACh-mediated neuromodulation, a deficit in cholinergic signaling is thought to contribute to the pathophysiology of various psychiatric illnesses that impair cognition, in particular schizophrenia (Higley and Picciotto, 2014). Moreover, mAChRs, specifically of the M1 subtype shown here to mediate many of the cholinergic effects in PFC, have been suggested as a potential target for schizophrenia treatment (Jones et al., 2012; Yohn and Conn, 2018). Alterations in γ oscillations and PV^+^ neurons in the PFC are a central feature in schizophrenia pathophysiology (Lewis, 2014). Thus, an interesting possibility is that abnormal M1 mAChR-mediated signaling (Scarr et al., 2018) is involved in the alterations in PV^+^ neurons, network oscillations, and cognition in schizophrenia.

## Synthesis

Reviewing Editor: Alfonso Araque, University of Minnesota Decisions are customarily a result of the Reviewing Editor and the peer reviewers coming together and discussing their recommendations until a consensus is reached. When revisions are invited, a fact-based synthesis statement explaining their decision and outlining what is needed to prepare a revision will be listed below. The following reviewer(s) agreed to reveal their identity: Allan Gulledge.

The reviewers have found merit and interest on the findings reported. I concur with the reviewers' comments expressing the interest of the study. However, they also expressed some concerns about several issues that need to be addressed and clarified. I also consider pertinent the concerns expressed. We believe that addressing those concerns, which may require less than 2 months' work and will definitely improve the manuscript, is necessary to firmly validate the results.

Specific comments of the reviewers:

Reviewer 1.

The present paper tests the impact of cholinergic signaling on the excitatory drive of different classes of fast-spiking interneurons in the medial prefrontal cortex (mPFC). The major strength of this study is the differentiation between three types of PV+ neurons, including basket and chandelier cells in L2, and basket cells in deeper layers. The main result is that activation of muscarinic ACh receptors increases the frequency and amplitude of AMPA-mediated EPSCs selectively in deep-layer basket neurons, having little effect on excitatory drive in layer 2 PV+ neurons. This seems consistent with the lamina-dependent effects of ACh on pyramidal neurons. The experiments are simple and straight forward, and unambiguously show differences in the cholinergic control of afferent excitatory input to these neuron classes. While limited in scope, these results are a step forward in our understanding of cholinergic regulation of cortical function.

Two main comments...

1) A limitation of this paper is that it does not report any tests of intrinsic excitability changes to ACh. Certainly changes in holding current or input resistance could be reported. Although changes in these values could result indirectly from barrages of EPSPs, they would at least somewhat address this important question... a question that the authors seem conflicted about, as in the abstract (see below) and introduction (line 67) and discussion (line 314), they cite papers suggesting direct effects of ACh on PV+ cell excitability, but in other places (lines 55 and 272) state that there is not direct excitation (citing the same papers, no less). This is a bit confusing, and should be clarified in the manuscript. It would help if the authors have any data from the present study that would address this issue.

2) The anatomical reconstructions are quite nice. One wonders whether those data can be more quantified, perhaps in table form, as to the mean extents of dendritic and axonal arborization, etc.

Minor comments...

The abstract's second line suggests that previous work shows that ACh regulates PV+ neuron excitability. But the authors later concede that this is not true in the mPFC (where the present study was performed). Thus, this line seems a bit misleading (see also comment 1, above).

Kawaguchi (1997) should probably be cited in lines 56 and 273.

Gulledge et al. (2007) should probably be cited in line 46.

On line 47, it might be useful to point out that the expression of nicotinic receptor appears to be dependent on cortical region (as per Hedrick & Waters, 2015).

One line 164, it would be nice to report the approximate thickness of L1 (~100 um?, 150 um?, smaller?).

Tsodyks & Markram (1997) and Levy et al. (2008) should probably be discussed near line 294.

The number of cells in Figure 2D, 4C, and 5C should be indicated, as the grey lines are hard to count.

Reviewer 2.

In the manuscript entitled 'Cell type- and layer-specific muscarinic potentiation of excitatory synaptic drive onto parvalbumin neurons in mouse prefrontal cortex, the authors analyze the effects of carbachol (CCh) in the spontaneous EPSCs (sEPSCs) recorded from PV+ neurons of the prefrontal cortex. The results demonstrate that CCh increases the frequency and amplitude of the sEPSCs of PV+ basket cells of Layer 3 to 6 being the sEPSCs from basket cells of layer 2 unaffected. Moreover, they show that CCh does not modulate the sEPSCs recorded from chandelier cells of layer 2 of the prefrontal cortex.

Understanding the cholinergic modulation of synaptic transmission at de PV+ neurons of prefrontal cortex is a significant topic and the manuscript does a nice job of characterizing the effect of CCH on sEPCs recorded from PV+ neurons of the prefrontal cortex. The experiments are well designed and provide convincing evidence for the increase in the synaptic drive to PV+ neurons of prefrontal cortex. However, there are few specific issues outlined below that should be addressed.

1. Data presented in the manuscript is pooled from neurons recorded from prelimbic, infralimbic and anterior cingulate regions of the prefrontal cortex. In my opinion the author must show that the results are no statistically different from PV+ neurons recorded in these different regions of the prefrontal cortex and how many cells have been recorded form these regions.

2. Although the effect of CCh is prevented by atropine (10microM) indicating that is mediated by muscarinic receptors, the authors should use some specific inhibitors of the M1 to M5 subtype of muscarinic receptors to determine the specific subtype involved in any case.

3. Although the authors analyzed the effect of CCH on the sEPSCs, In order to improve the quality of the paper it would be necessary to analyze whether the modulation is similar on the EPSCs evoked by stimulating nearby PCs.

4. In the same way, the manuscript would be significantly of better-quality by showing the effect of CCh on the miniature EPSCs recorded in the presence of TTX to show the CCh effect on the probability of release and not only excitability of the presynaptic pyramidal cells.

## Author Response

Dear Dr. Araque:

We are pleased to hear that our manuscript, “Cell type- and layer-specific muscarinic potentiation of excitatory synaptic drive onto parvalbumin neurons in mouse prefrontal cortex,” was found potentially suitable for publication in eNeuro, after a second round of revisions.

We have revised the manuscript to address the questions and concerns from Reviewer 1. We are submitting two new versions of the text, one with all changes indicated in red font, and a clean copy that includes the title page.

Below please find our point-by-point reply to the critiques from Reviewer 1. In our response, we indicate the location of the changes made using the page numbers in the version of the revised manuscript with the changes in red colored font.

We greatly appreciate the reviewers' feedback during the two rounds of revisions, which helped us significantly improve the manuscript. We hope our responses are found to be satisfactory, and that the revised manuscript will be suitable for publication in eNeuro.

Sincerely yours,

Reviewer 1

Main comments:

1) From Figure 1, it seems there is a wide expanse (&gt;200 um worth) of PFC lacking PV+ neurons. It is not clear whether this reflects a real bimodal distribution of PV+ cells with depth, or simply that the researchers did not test PV+ cells within those intermediate depths. This should be addressed somewhere in the text.

We thank the reviewer for helping us to clarify this section of the manuscript. Please, see our responses to the related comment 2 below, which highlights changes made in lines 209-229 of the revised version.

2) Line 226-232: It is really unclear what the authors mean here. What is the significance of “ greater than ~2.5 times the thickness of layer 1” if the border of 3 and 5 is defined as half the distance between the 1-2 border and white matter, while border of 5-6 was defined as halfway from the 3-5 border to the white matter. What is left to measure?

In response to this comment, we have extensively revised the corresponding text in the methods section to clarify the procedures we used to target cells for recording at different depths from the pial surface and to approximate the laminar boundaries relative to the locations of the recorded cells. See lines 209-229 of the revised version.

3) It may be worth noting that muscarinic excitation is more pronounced in deep-layer pyramidal neurons (e.g. Dembrow et al., 2010,, Joshi et al., 2016, or Baker et al., 2018), and since deep-layer basket cells likely get a larger fraction of their excitatory input from deep layer pyramidal neurons (as suggested in Figure 5C), this could go a long way toward explaining the difference in CCh effects in L2 vs L3-5 PV+ neurons.

We agree that muscarinic excitation is more pronounced in deep layer pyramidal neurons and made mention of that observation in several sections of the original manuscript. We also agree, as mentioned in the previous version of the manuscript, that the data in Figure 5C suggest that deep layer BCs receive a large fraction of input in deep layers. To emphasize these points further, we have added a few statements in lines 450-455.

4) On line 454, the authors state the CCh-induced depolarization of L3-6 BCs in the presence of CNQX and APV, as shown in Figure 11B, was “not significant”, although clearly there is something going on there. Here is where we see a limitation of interpreting data based on “p values”. The effect is so obvious, that the authors go on to test the pharmacology of this phenomena with atropine (Figure 11D). It is a conundrum. One wonders whether the experiment is under powered.

The main relevance of the experiments in Figure 11 is the contrast with the significantly stronger effect of CCh in pyramidal neurons (Figure 10). However, we agree with the reviewer that our conclusion that the subthreshold depolarization of PV+ cells by CCh is not significant (P=0.06) appears at odds with the visual impression from Figure 11B and seems inconsistent with the absence of change in Vm in the presence of atropine (Figure 11D). To resolve this apparent contradiction, we proceeded as follows. The original One Way RM ANOVA comparing baseline versus 5 and 8 minutes after CCh application indicated lack of significance, with post-hoc contrasts showing significant depolarization at 5 (p=0.024) but not 8 minutes (p=0.170). We repeated the statistical analysis, but now compared baseline and 5 minutes using paired sample t-test, as done for the pyramidal neuron data in Figure 10B. This comparison changed the outcome in two ways: first, the Shapiro Wilk normality test produced a non-significant value (p= 0.17); second, the effect of CCh was now significant (t(8)= 2.565, p=0.033). We believe this revision addresses the Reviewer's concern while not changing the take home message (depolarization by CCh is small in PV+ neurons compared to pyramidal cells). See revisions in the Statistics table and in lines 523-526 of the results section.

5) A related issue that should at least be discussed is that glutamate may still be working via unblocked NMDA receptors (e.g., the authors reference Bogart & O'Donnell, 2018) and/or metabotropic glutamate receptors, both of which would be stimulated by the increase in glutamate release even in the presence of CNQX, and could therefore generate depolarization of the PV+ neurons. These possibilities should be discussed in relation to Figure 11B.

We have mentioned the potential involvement of metabotropic and NMDA receptors in lines 564-567 and discussed the topic, including additional citations, in lines 716-726.

6) Also related to the above, line 236 states that error is measured as standard deviation or standard error of the mean “as indicated”. But is is not indicated in many places (it looks as if SD is never indicated). This should be looked at for accuracy.

All data in the revised version are expressed as mean {plus minus} SEM.

Minor:

1) Lines 184 and 191: stray periods.

This error has been corrected.

2) Line 192: “200-500 ms window preceding...” It is not clear what is driving spikes if they are measured before (presumably) depolarizing current steps.

We thank the reviewer for this comment, which helped us to clarify the main goal of this data analysis which was to test whether CCh-induced depolarization initiated spiking in pyramidal neurons independent of the injected current steps, in a manner consistent with increasing the EPSC frequency in PV+ BCs. However, anticipating the possibility that CCh neither caused depolarization nor initiated spikes but increased the response to excitatory current steps, our design included current steps. Given that CCh depolarized both pyramidal neurons and PV+ cells, by “default” CCh enhanced the response to current injection. However, we focused our analysis on the effects of CCh independent of the depolarizing current steps. To clarify these goals, we edited the text in the Methods section, see lines 160-168. We also clarified this issue in the text associated with Figure 10 (lines 457-459) and Figure 11 (lines 528-532). Please also see our response to minor comment 5) below.

3) Line 223; “thickness of layer thickness” ???

This error has been corrected. See lines 203-204.

4) Scale bars in Fig. 6A are different thicknesses. Better would be to rescale everything to a single scale bar (they look by eye to be fairly comparable in size), and to just have one scale bar for all of the morphologies. This would also be useful for other figures, as it allows direct comparison of sizes across neuron morphologies.

We have edited the Figures including neuron morphologies (Figures 2,3,4, and 6) to use a common single scale bar in each part of the figures and indicated this in each figure legend.

5) Figure 10: Panel C shows both Vm and spike frequency, and it is not clear why frequency is not shown in panel E. While panel E shows a slow and constant depolarization of the neuron, clearly something different is happening in C which has been blocked by the M1 antagonist. While the current steps used may have evoked higher frequency action potential generation as neurons depolarized with time, it seems likely that if neurons were held at their original resting membrane potential, that spike increases would only be observed in the absence of pirenzepine. If the authors have any data to address this, it could help reinforce the M1 component of the response.

As explained in our response to minor comment 2), the focus of the experiments in Figure 10 was to assess whether CCh initiated spiking by directly depolarizing the cells, because such an effect would help explain the potentiation of sEPSCs in BCs. We also monitored spiking produced by current steps, because we could not directly anticipate whether CCh would produce depolarization or not. However, given that CCh strongly depolarized the pyramidal neurons producing spiking, we did not assess changes in the response to injected current steps. To clarify this point, we added sentences in the text associated with Figure 10 (lines 457-459) and Figure 11 (lines 528-532). In Figures 10D and E we have added labels indicating time window of spike frequency measures even though in the presence of pirenzepine, carbachol did not initiate spikes.

6) Figure 11: The authors should state in the legend and, perhaps also the figure, that glutamate and GABA receptors were blocked. Also, a time course of spike frequency should be plotted, if available, as in Figure 10C.

We have added the mention to synaptic receptor blockers to Figures 10 and 11 and to the figure legends. We have added labels indicating the time window of spike measurements, as in Figure 10.
